# Deciphering the suppressive immune microenvironment of prostate cancer based on CD4+ regulatory T cells: Implications for prognosis and therapy prediction

**DOI:** 10.1002/ctm2.1552

**Published:** 2024-01-18

**Authors:** Qintao Ge, Zhijie Zhao, Xiao Li, Feixiang Yang, Meng Zhang, Zongyao Hao, Chaozhao Liang, Jialin Meng

**Affiliations:** ^1^ Department of Urology The First Affiliated Hospital of Anhui Medical University Hefei P. R. China; ^2^ Institute of Urology Anhui Medical University Hefei P. R. China; ^3^ Anhui Province Key Laboratory of Urological and Andrological Diseases Research and Medical Transformation Anhui Medical University Hefei P. R. China; ^4^ Department of Plastic and Reconstructive Surgery, Shanghai Ninth People's Hospital Shanghai Jiao Tong University School of Medicine Shanghai P. R. China; ^5^ Anhui Provincial Institute of Translational Medicine Hefei P. R. China

Dear Editor,

Prostate cancer (PCa) is traditionally considered an immunologically cold tumour characterized by an immunosuppressive tumour microenvironment (TME) and a disappointing response to immunotherapy. Nevertheless, recent studies have demonstrated that the TME of PCa is heterogeneous, and some PCa patients still exhibit “hot tumours” and are sensitive to immunotherapy.^1^ Our prior works also classified PCa into three phenotypes according to variable immune status and emphasized the important role of regulatory T (Treg) cells in shaping the exhausted TME in PCa.^2^ In this study, we further conducted an in‐depth discussion on Treg cells, and the detailed methods are listed in the Supporting Information Data.

A total of 56924 cells from 14 samples with Gleason scores were clustered and annotated into 13 cell types, where the high variable of T cell attracted much attention (Figure 1A–C and Figure S1). We extracted and re‐clustered all T cells into 11 clusters (Figure S2A). Referring to published articles, these cells were annotated into six cell populations (Figure 1D–F and Figure S2B), where C3 exhibited the highest Treg score (*p* < 2.2e‐16) and C5 had the highest Th17 score (*p* < 2.1e‐16); we annotated C3 as Treg cells and C5 as Th17 cells (Figure 1G). The ratio of Treg and Th17 is positively correlated with Gleason groups (*p* = .04, R = 0.65; Figure 1H and Figure S2C), which was also comfited in an external dataset (HRA000823, *p* = .039, R = 0.56; Figure S3), indicating a positive correlation between high Treg infiltration and poor prognosis. In multiplex immunofluorescence experiment, more CD4+FOXP+Treg cells were observed in high‐risk PCa samples (Gleason score 4+5, PSA > 100 ng/dL, T3bN1M0) than in low‐risk samples (Gleason score 3+4, PSA 25.39 ng/dL, T2N0M0) (Figure 1I). In addition, the abundances of CD8+effector, CTL, activate B and plasma B cells increased with Gleason groups, and SPP1+macrophage presented a positive correlation with Gleason groups, while no significance showed (Figures S4–S6). Trajectory analysis revealed Treg and Th17 differentiated in distinct directions (Figure 2A), and the expression patterns of branch‐dependent genes were different. As shown in Figure 2B, G3 genes increased as cells differentiated toward branch 2 (Treg) and were enriched in the negative regulation of immune system processes, which might better represent mature Treg cells.

**FIGURE 1:**
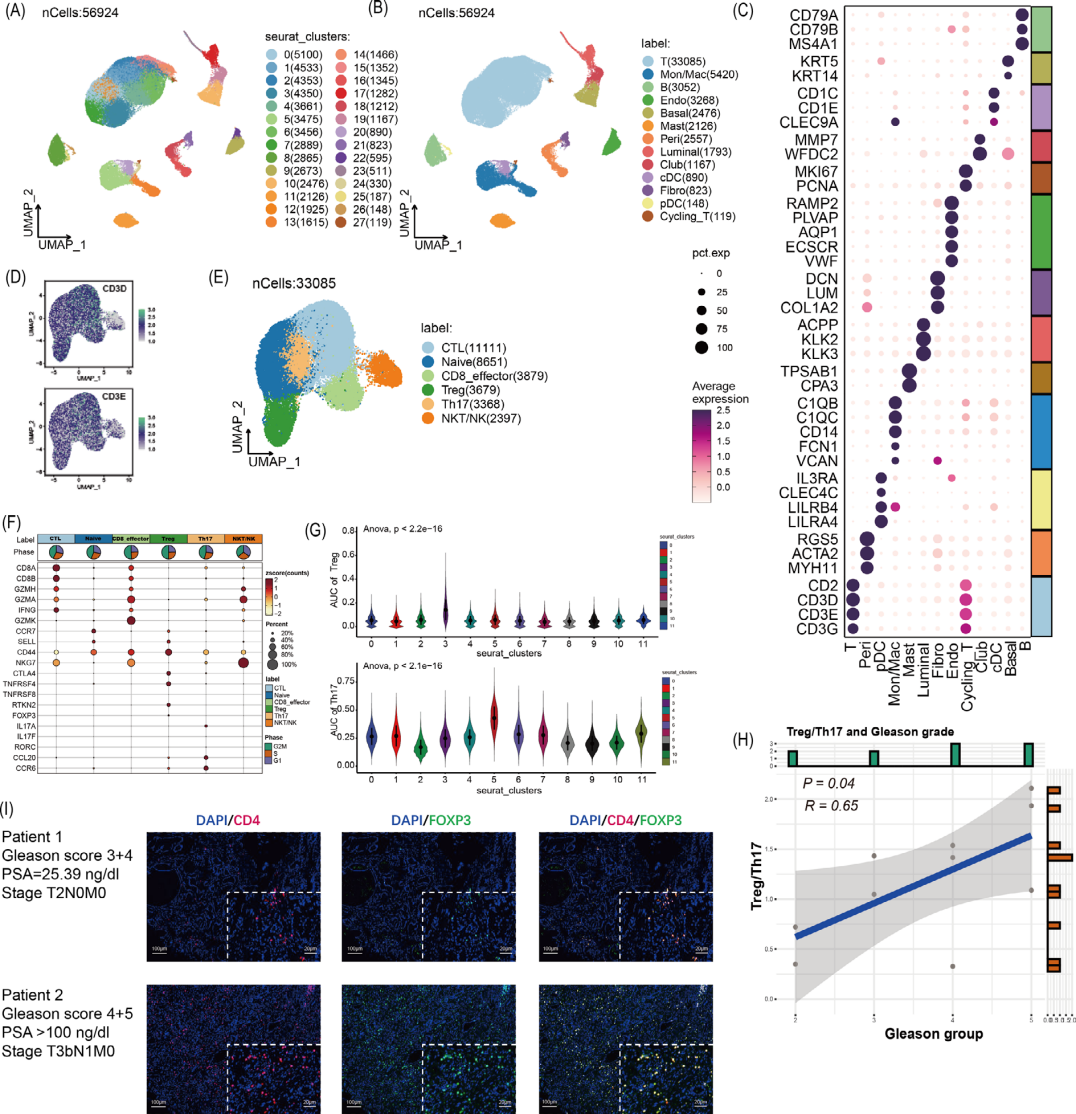
Single‐cell analysis. (A) Uniform manifold approximation and projection (UMAP) visualization of 28 clusters. (B) UMAP visualization of 13 cell types, including 33085 T cells, 3052 B cells, 3268 endothelial cells, 2476 basal epithelial cells, 1793 luminal epithelial cells, 1167 club epithelial cells, 2126 mast cells, 2557 pericytes, 890 cDC cells, 148 pDC cells, 823 fibroblasts, 5420 macrophages/monocytes and 119 cycling T cell. (C) Dot plot showing the expression level of marker genes, including T cells (*CD2, CD3D* and *CD3E*), B cells (*CD79A, CD79B* and *MS4A1*), endothelial cells (*RAMP2, PLVAP, AQP1, ECSCR* and *VWF*), basal epithelial cells (*KRT5* and *KRT14*), luminal epithelial cells (*ACPP, KLK2* and *KLK3*), club epithelial cells (*MMP7* and *WFDC2*), mast cells (*TPSAB1* and *CPA3*), pericytes (*RGS5, ACTA2* and *MYH11*), cDC cells (*CD1C, CD1E* and *CLEC9A*), pDC cells (*IL3RA, CLEC4C, LILRB4* and *LILRA4*), fibroblasts (*VWF, DCN, LUM*), macrophages/monocytes (*C1QB, C1QC, CD14, FCN1* and *VCAN*) and cycling T cells (*CD2, CD3D, CD3E, MKI67* and *PCNA*). (D) A total of 33,085 T cells were further extracted and performed batch effect elimination, dimensionality reduction and clustering and the UMAP plot showed the expression of specific T cell markers of *CD3D* and *CD3E*. (E) The UMAP showed six types of T cells (11111 CTL, 3879 CD8^+^ T effector, 2397 NKT cells, 8651 CD4^+^ naïve T, 3679 CD4^+^ regulatory T [Treg] and 3368 Th17 cells). (F) Dot plot showing the expression of specific T cell markers among these six type T cells, including CTL (*CD8A, CD8B, GZMH* and *GZMA*), CD8+ T effector cells (*CD8A, CD8B, IFNG* and *GZMK*), NKT cells (*NKG7* and *CD44*), CD4+ naïve T cells (*CCR7* and *SELL*), CD4+ Treg cell (*CTLA4, TNFRSF4, TNFRSF8, RTKN2* and *FOXP3*) and Th17 cells (*IL17A, IL17F, RORC, CCL20* and *CCR6*). (G) Comparison of the AUCell score of Treg activity between the C3 cluster and the other 10 clusters (upper); Comparison of the AUCell score of Th17 activity between the C5 cluster and the other 10 clusters (lower). (H) The correlation between Gleason groups and Treg/Th17 ratios. (I) Multiplex immunofluorescence showed the location and co‐location of CD4 and FOXP3 in prostate cancer (PCa) tissues.

**FIGURE 2:**
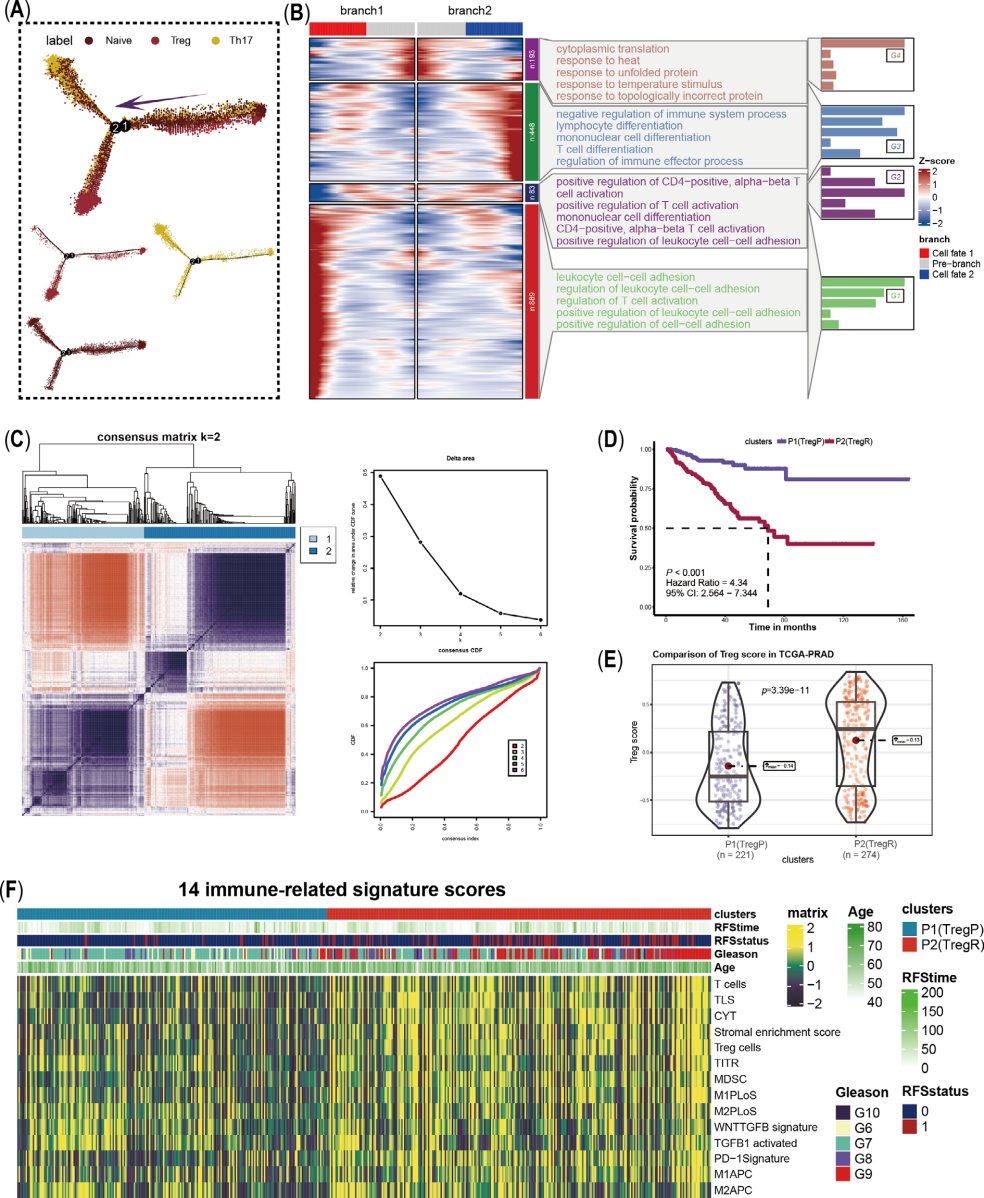
Pseudotime analysis of CD4+T cells and construction of regulatory T (Treg)‐prostate cancer (PCa) classification. (A) Differentiation trajectories of CD4+Treg, Th17 and CD4+Naïve cells. (B) Branch point analysis based on pseudotime trajectory and GO enrichment analysis. Genes of C1 expressed increase significantly as cells differentiate towards branch1 (Th17), and enriched into positive regulation of immune pathways; less difference of C2 genes was observed in the two cell fates, which were more likely related to positive regulation of T cell activation, mononuclear cell differentiation and positive regulation of leukocyte cell‐cell adhesion; genes of C3 increases as cells differentiate towards branch 2 (Treg), and were enriched into negative of immune system process; C4 genes highly expressed at the initiation of two cell fates, which were enriched to cytoplasmic translation, response to heat, response to unfolding protein, response to temperature stimulus and response to topologically incorrect protein. (C) Construction of a binary classification system for PCa based on unsupervised clustering algorithms and 75 branch‐dependent genes. (D) K‐M plot showed poor‐Treg PCa (TregP) had a higher survival probability than rich‐Treg PCa (TregR). (E) Comparison of Treg score between TregP and TregR. (F) Distribution of 14 immune‐relevant signatures among TregP and TregR.

Based on branch‐dependent genes in the G3 cluster (Figures S7 and S8), we assigned patients in the TCGA‐PRAD cohort into two phenotypes (Figure 2C): P2 exhibited a poorer prognosis (*p* < .001, hazards ratio [HR] = 4.34, 95% confidence interval [CI]: 2.564–7.344), and a higher Treg activity score (*p* = 3.39e‐11), thereby nomenclature as rich‐Treg PCa (TregR) and P1 as poor‐Treg PCa (TregP) (Figure 2D,E). Notably, the two groups exhibited distinct inflammation and immune status (Figure S9). Compared to TregP, TregR had higher scores for immune‐suppressed signatures, such as transforming growth factor‐β (TGF‐β), myeloid‐derived suppressor cell (MDSC), tertiary lymphoid structure (TLS) and programmed cell death 1 (PD‐1) signatures (Figure 2F). Interestingly, higher scores of these immune‐suppressed signatures were also observed in the immune‐suppressed subtype, where TGF‐β might be the pivotal enforcer of immune tolerance and homeostasis. Peripheral CD4+ T cells reportedly produce TGF‐β under suboptimal stimulation, initiating Treg transformation in malignant cells.^3^ Treg cells also secrete abundant TGF‐β, inhibiting CD8+ T cells and thus advancing tumour progression.^4^ Jiao et al. also emphasized the essential role of TGF‐β in shaping suppressive microenvironment in PCa. As a common result, immunotherapy combined with TGF‐β inhibition may be a better treatment option to further improve survival rates.^5^

In the three external cohorts, the risk of recurrence was more than 6‐fold greater for TregR than for TregP in the real‐world AHMU‐PC cohort (*p* < .001) and 2‐fold greater in the MSKCC (*p* = .026) and GSE23136 (*p* = .026) cohorts (Figure 3). Similar trends, with elevated TGF‐β, MDSC, TLS and PD‐1 scores in the TregR subtype, were consistent across all cohorts (Figure S10).

**FIGURE 3:**
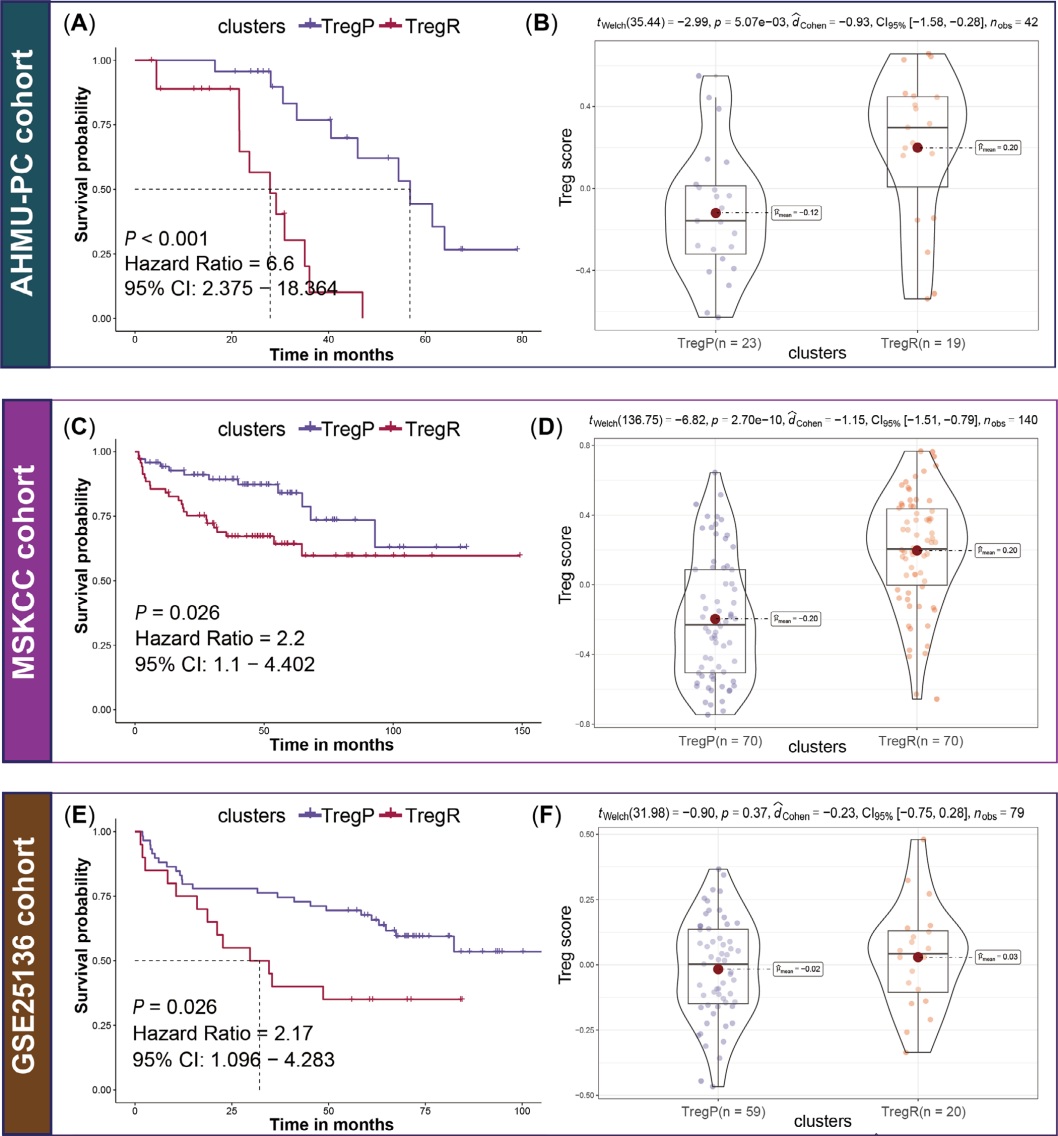
Validation in three external cohorts. (A) K‐M analysis in AHMU‐PC cohort. Rich‐Treg PCa (TregR) presented shorter recurrence‐free survival (RFS, *p* < .001, hazards ratio [HR] = 6.6, 95%CI: 2.375–18.364) than poor‐Treg PCa (TregP); (B) Comparison of the Treg activity between TregP and TregR in AHMU‐PC cohort, TregR presented higher Treg score (*p* = 5.07e‐03) than TregP; (C) K‐M analysis in MSKCC cohort. Compared to TregP, TregR presented a shorter RFS (*p* = .026, HR = 0.45, 95%CI: 0.227–0.909); (D) Comparison of the Treg activity between TregP and TregR in MSKCC cohort, and TregR presented higher Treg score (*p* = 2.70e‐10) than TregP; (E) K‐M analysis in GSE25136 cohort. TregR had poorer clinical outcome than TregP (*p* = .026, HR = 2.17, 95%CI: 0.096–4.283); (F) Comparison of the Treg activity between TregP and TregR in the GSE25136 cohort, higher Treg score was observed in TregR despite no statistical significance (*p* = .37), which might be related to the small sample size.

Multiple somatic nucleotide variations underlie the high heterogeneity of PCa, and TregR and TregP showed distinct mutation landscapes (Figure S11 and Table S4), among which the mutational differences in *TP53* and *PIK3CA* attracted our attention (Figure 4A). Variants of the *TP53* gene predisposed patients to aggressive PCa, elevated chemoresistance and poor sensitivity to anti‐PD‐1 therapy,^6^, ^7^, ^8^ which might be one of the explanations for limited available treatments for TregR. Among the enrolled eight chemicals, only cisplatin (*p* = .004) and 5‐fluorouracil (*p* = .028) were effective against TregR, while bicalutamide (*p* = 4.2e‐11), doxorubicin (*p* = 5.3e‐06), etoposide (*p* = 1.9e‐10), gemcitabine (*p* = .00053), mitomycin C (*p* = .0021) and vinorelbine (p = 2e‐08) were better for TregP (Figure 4B), as well as anti‐PD‐1 therapy (Figure 4C). Overall, the susceptibility of the two subtypes to the eight drugs was broadly consistent across the three cohorts, except for cisplatin (Figure S12). As an upstream effector of the PI3K‐AKT‐mTOR pathway, PI3K activation usually occurs in advanced tumours and *PI3KA* mutations are observed among nearly 28%–30% of castration‐resistant PCa.^9^ PI3K‐AKT‐mTOR pathway may be aberrantly activated when AR receptors are strongly inhibited, which contributes to the poor response of TregR cells to bicalutamide.^10^ On the other hand, PI3K inhibitors such as BKM120 and PX966 may help restore sensitivity to bicalutamide in TregR.

**FIGURE 4:**
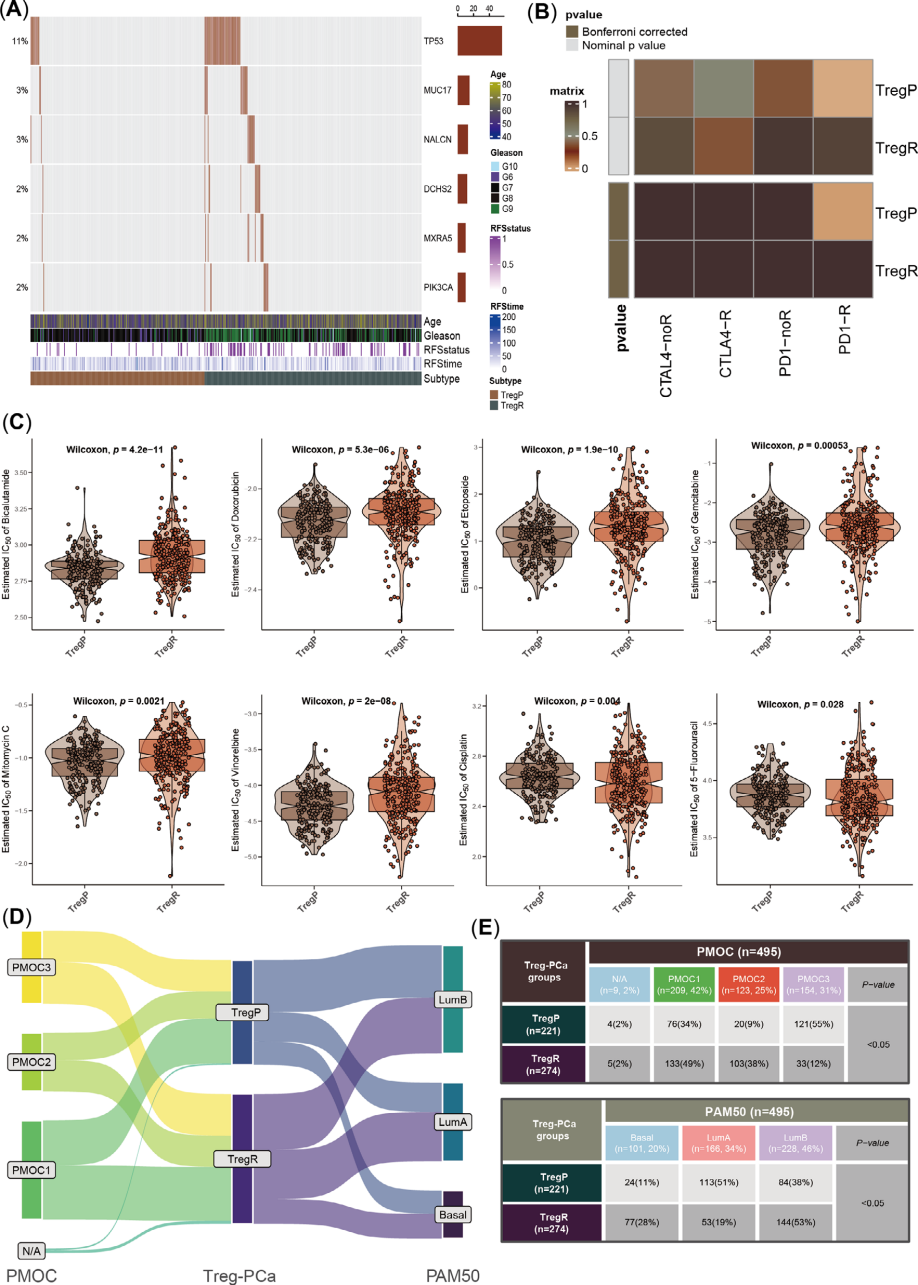
Comparison of gene mutation patterns, sensitivity to immunotherapy and chemotherapy and published molecular classifications. (A) The top six most differently mutated genes between rich‐Treg PCa (TregR) and poor‐Treg PCa (TregP), including *TP53, MUC17, NALCN, DCHS2, DCHS2, MXRA5* and *PIK3CA*. (B) SubMap analysis for deciphering the different responses to anti‐PD‐1 or CTLA‐4 therapy. (C) Comparison of the sensitivity to eight chemotherapeutic drugs, including bicalutamide, doxorubicin, etoposide, gemcitabine, mitomycin C, vinorelbine, cisplatin, 5‐fluorouracil and the lower IC50, the higher the sensitivity. (D) Sankey plot showed the distributions of TregP and TregR in PMOC classification (left) and PAM50 classification (right). (E) χ2 test table showed the concrete proportions of TregP and TregR subtypes in different molecular classifications.

We further compared PCa‐Treg classification with two proposed classifications (Figure 4D); the detailed information was listed in Supplementary Methods. more TregR belonged to PMOC2, with a weak response to androgen deprivation therapy (ADT) and higher mutation frequency of *TP53*, resulting in poor prognosis, which was also consistent with TregR (Figure 4E). The overlap of TregR/PMOC2 defined the worst phenotype (*p* < .0001, Figure S13A). In addition, more TregRs belonged to the luminal B subtype than to the TregP subtype (Figure 4E), and the TregR/luminal B group also exhibited the poorest phenotype (*p* < .0001; Figure S13B). Similar clinical features of TregR, PMOC2 and luminal B indicated the stability of molecular typing to some extent. Although high‐risk PCa is determined by many factors, Tregs are heavily weighted.

In this study, we combined single‐cell and bulk RNA sequencing to verify the driving role of Tregs in PCa. Two phenotypes were defined, where TregR represented a poor prognosis, immunosuppressed and poor therapeutic response phenotype and TregP was the opposite. Characteristics such as TGF‐β expression and PI3KA and TP53 mutations facilitate the resistance to anti‐PD‐1 therapy or ADT, further studies are needed to explore the specific underlying mechanisms, which will help us overcome ADT resistance and reverse the “cold” TME in TregR PCa.

## AUTHOR CONTRIBUTIONS

Conceptualization, QG, ZZ and XL; methodology, QG, ZZ, XL and JM; formal analysis, QG, FY and JM; investigation, QG, ZZ, MZ, JM, XL and CL; writing the original draft, QG, JM and ZZ; visualization, QG and ZZ; funding acquisition, ZH and JM; supervision, ZH, JM and CL.

## CONFLICT OF INTEREST STATEMENT

The authors declare no conflict of interest.

## FUNDING INFORMATION

This work was supported by the Anhui Province Key Project for Clinical Medical Research Translation and Advancement (202204295107020031).

## ETHICS STATEMENT

Ethical approval for this study was obtained from the Ethics Committee of the First Affiliated Hospital of Anhui Medical University (approval number: PJ‐2019‐09‐11).

## Supporting information

Supporting Information

## Data Availability

The raw data for this study were generated at the corresponding archives, further inquiries can be directed to the corresponding authors.
