# Supplementary material for: Deciphering the suppressive immune microenvironment of prostate cancer based on CD4+ regulatory T cells: Implications for prognosis and therapy prediction
Source: Clin Transl Med. 2024 Jan 18;14(1):e1552. doi: 10.1002/ctm2.1552 (PMC10797244; doi:10.1002/ctm2.1552)
Supplement: Supplementary file 1 — Supporting Information [file CTM2-14-e1552-s001.docx]

**Deciphering the suppressive immune microenvironment of prostate cancer based on CD4+ Regulatory T cell: implication for prognosis and therapy prediction**

**SUPPLEMENTARY MATERIALS AND METHOD ……………………Page 2**

**SUPPLEMENTARY TABLES S1-4 …………………………………….…Page 8**

**SUPPLEMENTARY FIGURES S1-13 ………………………………….…Page 17**

**Materials and method**

**Row data collection**

We searched single-cell RNA-sequencing data of PCa in Gene Expression Omnibus (GEO, <https://www.ncbi.nlm.nih.gov/geo/>) and enrolled GSE181294 cohort. Fourteen tumor samples with corresponding Gleason score parameters were eventually enrolled in the following analysis, the 2014 international society of urological pathology grade group classification (GG) was also used to present the degree of malignancy of the 14 patients(1), and the detail information was showed in **Table S1**. To test the results in GSE181294, we obtained a pre-processed external single-cell RNA-sequencing from the Genome Sequence Archive (GSA, <https://ngdc.cncb.ac.cn/gsa-human/>), with the accession number HRA000823. Bulk sequencing profiles and matched clinicopathological data was used for further analysis, where TCGA-PRAD (n=495) cohort was from GDC platform (<https://portal.gdc.cancer.gov/>), MSKCC (n=140) cohort was from MSKCC Prostate Cancer Oncogenome Project ([https://cbio.mskcc.org/](https://cbio.mskcc.org/cancergenomics/prostate/data) ), GSE25136 (n=79) cohort was from GEO platform. In addition, a real-world AHMU-PC cohort which contained 69 sample was enrolled, and further filtrated referring to local mean age to exclude the influence of natural death on the prognosis, and 42 patients were eventually enrolled for further study. Recurrence-free survival (RFS) was regarded as the index to evaluate prognosis of PCa patients. All of the expression data was annotated by corresponding platform and expressed as Transcripts Per Million (TPM), and the detail baseline information showed in **Table S2**.

**Preprocessing of single-cell RNA-seq data**

For enrolled 10x data, the R package “Seurat” (2) was used for preprocessing. Double cells were removed using the R package “DoubletFinder”(3) and inferior cells were filtered. Exclusion criteria: 1) per cell with detected a gene number > 6000 or <200; 2) The proportion of mitochondrial gene count more than 20%. As for gene filtration, those expressed in less than five cells were excluded. The function CellCycleScoring () was performed to evaluate the cell cycle status, and then using regressout algorithm in ScaleData() function to remove cell cycle effects. After normalizing and scaling the expression data, batch effect was eliminated through the R package “Harmony”, and the top 2000 variable genes were identified. Principal component analysis was used for dimensionality reduction based on the 2000 variable genes. The distances in the first 15 principal components are used to create the UMAP embedding. With a resolution of 1.2, cell clustering was performed using FindClusters () function. The annotation of clustered cell population was performed manually based on the signature genes of different cell types, referring to the published literatures(4, 5). According to Ruben Bill et al., traditional monoculture schemes that distinguish M1 and M2 macrophages were inferior to SPP1 and CXCR9, which would better reflect the macrophage polarity and prognosis difference(6). In present study, we used combined signature genes and top Differentially expressed genes (DEGs) to annotate different cell populations. The expression of SPP1 and CXCR9 among different macrophages were also concerned. Unfortunately, the expression of CXCR9 in each cell is extremely low, and only the results of SPP1 are presented.

**Calculation of Treg activity, Th17 activity, T cell cytotoxicity and M2 macrophage scores.**

AUCell allows to calculate the enrichment score of given gene sets in single-cell RNA-seq data (7). We define the top quantile as the top 5% of gene signatures. The gene set (*CTLA4, SOD1, TNFRSF4, TNFRSF18, RTKN2, FOXP3, TIGIT, CTLA4*), the gene set (*IL17A, IL17F, RORC, CD4, CCL20, CCR6, RORA*), the gene set (*GZMA, GZMB, GZMM, GZMK, PRF1, CD8A, CD8B*), the gene set (*ARG1, ARG2, IL10, CD32, CD163, CD23, CD200R1, PD-L2, PD-L1, MARCO, CSF1R, CD206, IL1RA, IL1R2, IL4R, CCL4, CCL13, CCL20, CCL17, CCL18, CCL22, CCL24, LYVE1, VEGFA, VEGFB, VEGFC, VEGFD, EGF, Cathepsin A, CTSB, CSTC, CTSD, TGFB1, TGFB2, TGFB3, MMP14, MMP19, MMP9, CLEC7A, WNT7b, FASL, TNFSF12, TNFSF8, CD276, VTCN1, MSR1, FN1, IRF4*) were used to calculate the activities of Treg, Th17 cells, and T cell cytotoxicity scores(4).

**Pseudo-time series analysis**

After annotation, we extracted all of the objects of CD4+T cells to enter pseudo-time series analysis using the R package “monocle2” to reveal the cell state transition(8). The dispersionTable() function was used to select genes for trajectory inference via calculating a smooth function which describes how the variance of each gene's expression varies with respect to the average, the “DDRTree” algorithm was used to reduce data dimensionality, and reduceDimension() function was employed to calculate variable differentiation state, and plot_cell_trajectory() was used for visualization. Branched expression analysis modeling (BEAM) was performed to identify genes with branch-dependent expression, and was visualized via plot_genes_branched_heatmap2() function in “ClusterGVis” package, as well as relevant enriched pathways.

**Unsupervised analysis and PCa-Treg classification establishment**

For genes that differ between the branches, univariate Cox regression analysis was further conducted in TCGA-PRAD cohort to filtrate prognostic genes as input into unsupervised algorithm in “ConsensusClusterPlus” package(9)(**Fig. S7**). By taking the intersection, we identified the input genes of MSKCC, GSE25136, and AHMU-PC cohorts, which were listed in **Table S3**. We set the parameters of unsupervised algorithm as follows: 50 repeats, *k*_max_ = 10, and agglomerative hierarchical clustering with Pearson distance. Based on the consistency within the cluster, variation in coefficient, and the increase in the area under the consensus cumulative distribution (CDF) curve, the optimal number of clusters was selected. All of the patients were assigned into two clusters, and K-M plot was used to described their prognosis difference, and the distribution of model-construction genes were showed in **Fig. S8**. Using the gsva algorithms in “GSVA” package and Treg signature genes, we calculated and compared Treg scores among the two clusters. Meanwhile, 25 immune relevant gene sets that represent different immune features was employed to illustrated the distinct immune status(10).

**Pathways enrichment analysis and gene mutation analysis**

DEGs between the two subtypes were selected with the threshold of | log FC | > 1 and *p* < 0.05, and further annotated referring to GO, KEGG, HALLMARK pathways, which was performed by using R package “clusterProfiler”(11), and *P*-values were adjusted for multiple testing using the Benjamini-Hochberg method with a threshold of FDR <0.05. We used the compMut module in R package “MOVICS”(12) to decipher the gene mutation frequency of the two subtypes, compTMB module to explore the tumor mutation burden (TMB), and compFGA module to elucidate the features of fraction of genome altered (FGA), fraction of genome loss (FGL), fraction of genome gained (FGG).

**Prediction of immunotherapy and chemotherapy**

Referring to the drugs from GDSC2016 (<https://www.cancerrxgene.org/>), and drugs that commonly used in PCa, we calculated and compared the estimated inhibitory concentration (IC50) for the two subtypes to filtrate worth-trying chemotherapeutic agents for patients with distinct CD4+ Treg cell infiltration, and the compDrugsen module in “MOVICS” was used to performed such program. Besides, we detected the similarity of gene expression profile between PCa and responders of anti-CTLA-4 or anti-PD-1 in the melanoma cohort based on the SubMap item in GenePattern, thereby identifying sensitive patient to receive immunotherapy.

**Comparison of newly defined subtypes with other molecular classifications**

We also explored the distribution of TCGA-PRAD patients in two published molecular classifications, including PAM50 classification and PMOC multi-omics classifications. K-M plot was employed to evaluate their prognostic characteristics. PAM50 defined PCa into luminal A, luminal B, and basal subtypes, where luminal B represented the poorest PCa(13); In previous studies, we exploited “MOVICS” algorithms to integrate multi-omics data and clustering, the multi-omics features which included mRNA, microRNA, long noncoding RNA, DNA methylation, and somatic mutation of PCa and divided PCa into PMOC1, PMOC2, and PMOC3 clusters, where PMOC2 indicated the most malignant phenotype(14). K-M plot was employed to evaluate their prognostic characteristics.

**Multiplex immunofluorescence assay**

We employed multiplex immunofluorescence (mIF) to investigate the heterogenous abundance of Treg cell among different PCa. Tissues for mIF was taken from previously collected paraffin-embedded surgical specimens from Pca patients who had signed an informed consent form (Ethics approval was obtained from the Ethics Committee of the First Affiliated Hospital of Anhui Medical University: PJ‐2019‐09‐11), and the final results presented are from a high-risk patient (Gleason score 3+4, PSA 25.39 ng/dl, T2N0M0) and a low-risk patient (Gleason score 4+5, PSA >100 ng/dl, T3bN1M0). CD4 and FOXP3 were employed to identify CD4+FOXP3+ Treg cells. Primary antibodies were stained using the specific antibodies of anti-CD4 (Abcam, Catalog: ab288724), anti-FOXP3 (Abcam, Catalog: ab215206), with the dilution multiple of 1:1000. Subsequently, the TSA-570 fluorescence dye (1:500) binding to CD4, which can be observed with red fluorescence; the TSA-520 (1:500) fluorescence dye binding to FOXP3, which can be observed with green fluorescence; and cell nuclei were stained with DAPI, and can be observed with blue fluorescence.

**Statistical analysis**

The survival outcomes were compared by the log-rank test, the categorical data were analyzed via Fisher’s exact test and rank sum test. T -test was used for comparison between two groups, while ANOVA was used for pairwise comparison among multiple groups. All statistical analyses were performed using R (Version: 4.2.2). A two-tailed *p*-value < 0.05 was recognized as statistically significant.

***Reference***

1. Epstein JI, Egevad L, Amin MB, Delahunt B, Srigley JR, Humphrey PA. The 2014 International Society of Urological Pathology (ISUP) Consensus Conference on Gleason Grading of Prostatic Carcinoma: Definition of Grading Patterns and Proposal for a New Grading System. Am J Surg Pathol. 2016;40(2):244-52.

2. Hao Y, Hao S, Andersen-Nissen E, Mauck WM, 3rd, Zheng S, Butler A, et al. Integrated analysis of multimodal single-cell data. Cell. 2021;184(13):3573-87.e29.

3. McGinnis CS, Murrow LM, Gartner ZJ. DoubletFinder: Doublet Detection in Single-Cell RNA Sequencing Data Using Artificial Nearest Neighbors. Cell Syst. 2019;8(4):329-37.e4.

4. Hirz T, Mei S, Sarkar H, Kfoury Y, Wu S, Verhoeven BM, et al. Dissecting the immune suppressive human prostate tumor microenvironment via integrated single-cell and spatial transcriptomic analyses. Nat Commun. 2023;14(1):663.

5. Zhao Z, Ding Y, Tran LJ, Chai G, Lin L. Innovative breakthroughs facilitated by single-cell multi-omics: manipulating natural killer cell functionality correlates with a novel subcategory of melanoma cells. Front Immunol. 2023;14:1196892.

6. Bill R, Wirapati P, Messemaker M, Roh W, Zitti B, Duval F, et al. CXCL9:SPP1 macrophage polarity identifies a network of cellular programs that control human cancers. Science. 2023;381(6657):515-24.

7. Aibar S, González-Blas CB, Moerman T, Huynh-Thu VA, Imrichova H, Hulselmans G, et al. SCENIC: single-cell regulatory network inference and clustering. Nat Methods. 2017;14(11):1083-6.

8. Qiu X, Hill A, Packer J, Lin D, Ma YA, Trapnell C. Single-cell mRNA quantification and differential analysis with Census. Nat Methods. 2017;14(3):309-15.

9. Wilkerson MD, Hayes DN. ConsensusClusterPlus: a class discovery tool with confidence assessments and item tracking. Bioinformatics. 2010;26(12):1572-3.

10. Meng J, Zhou Y, Lu X, Bian Z, Chen Y, Zhou J, et al. Immune response drives outcomes in prostate cancer: implications for immunotherapy. Mol Oncol. 2021;15(5):1358-75.

11. Yu G, Wang LG, Han Y, He QY. clusterProfiler: an R package for comparing biological themes among gene clusters. Omics. 2012;16(5):284-7.

12. Lu X, Meng J, Zhou Y, Jiang L, Yan F. MOVICS: an R package for multi-omics integration and visualization in cancer subtyping. Bioinformatics. 2020.

13. Zhao SG, Chang SL, Erho N, Yu M, Lehrer J, Alshalalfa M, et al. Associations of Luminal and Basal Subtyping of Prostate Cancer With Prognosis and Response to Androgen Deprivation Therapy. JAMA Oncol. 2017;3(12):1663-72.

14. Meng J, Lu X, Jin C, Zhou Y, Ge Q, Zhou J, et al. Integrated multi-omics data reveals the molecular subtypes and guides the androgen receptor signalling inhibitor treatment of prostate cancer. Clin Transl Med. 2021;11(12):e655.

**SUPPLEMENTARY TABLES**

**Table S1.** The detail information of the 14 samples with single-cell sequencing data.

| **Projects** | **Sample id** | **Age** | **Grade** | **Gleason score** | **Gleason group*** |
| --- | --- | --- | --- | --- | --- |
| G331 | GSM5494362 | 46 | Low grade | 3+3=6 | GG1 |
| G332 | GSM5494365 | 54 | Low grade | 3+3=6 | GG1 |
| G333 | GSM5494371 | 65 | Low grade | 3+3=6 | GG1 |
| G334 | GSM5494372 | 59 | Low grade | 3+3=6 | GG1 |
| G341 | GSM5494348 | 73 | Low grade | 3+4=7 | GG2 |
| G342 | GSM5494350 | 69 | Low grade | 3+4=7 | GG2 |
| G431 | GSM5494354 | 68 | Low grade | 4+3=7 | GG3 |
| G432 | GSM5494360 | 65 | Low grade | 4+3=7 | GG3 |
| G351 | GSM5494356 | 57 | High grade | 3+5=8 | GG4 |
| G441 | GSM5494359 | 66 | High grade | 4+4=8 | GG4 |
| G531 | GSM5494374 | 59 | High grade | 5+3=8 | GG4 |
| G451 | GSM5494352 | 71 | High grade | 4+5=9 | GG5 |
| G452 | GSM5494369 | 61 | High grade | 4+5=9 | GG5 |
| G551 | GSM5494358 | 53 | High grade | 5+5=10 | GG5 |

***** The 2014 international society of urological pathology grade group classification (GG)

**Table S2.** The baseline profiles of the 756 patients with bulk RNA-sequencing data.

|  | **AHMU-PC (N=42)** | **GSE25136 (N=79)** | **MSKCC (N=140)** | **TCGA-PRAD (N=495)** | **Overall (N=756)** |
| --- | --- | --- | --- | --- | --- |
| **RFStime** |  |  |  |  |  |
| Mean (SD) | 32.5 (18.6) | 51.5 (30.4) | 46.0 (30.3) | 31.5 (24.8) | 36.4 (27.3) |
| Median [Min, Max] | 28.2 [3.38, 79.0] | 61.1 [1.40, 106] | 45.5 [1.38, 149] | 25.8 [0.750, 165] | 30.5 [0.750, 165] |
| **RFSstatus** |  |  |  |  |  |
| No | 20 (47.6%) | 42 (53.2%) | 104 (74.3%) | 402 (81.2%) | 568 (75.1%) |
| Yes | 22 (52.4%) | 37 (46.8%) | 36 (25.7%) | 93 (18.8%) | 188 (24.9%) |
| **Age** |  |  |  |  |  |
| Mean (SD) | 66.4 (6.70) | 60.6 (6.18) | 58.1 (6.97) | 61.0 (6.84) | 60.7 (7.00) |
| Median [Min, Max] | 69.0 [49.0, 73.0] | 61.2 [44.9, 72.7] | 58.0 [37.3, 83.0] | 61.0 [41.0, 78.0] | 61.0 [37.3, 83.0] |
| **Gleason** |  |  |  |  |  |
| 10 | 2 (4.8%) | 0 (0%) | 0 (0%) | 4 (0.8%) | 6 (0.8%) |
| 6 | 10 (23.8%) | 17 (21.5%) | 41 (29.3%) | 45 (9.1%) | 113 (14.9%) |
| 7 | 13 (31.0%) | 44 (55.7%) | 76 (54.3%) | 246 (49.7%) | 379 (50.1%) |
| 8 | 8 (19.0%) | 10 (12.7%) | 11 (7.9%) | 63 (12.7%) | 92 (12.2%) |
| 9 | 9 (21.4%) | 8 (10.1%) | 10 (7.1%) | 137 (27.7%) | 164 (21.7%) |
| unknow | 0 (0%) | 0 (0%) | 2 (1.4%) | 0 (0%) | 2 (0.3%) |
| **T stage** |  |  |  |  |  |
| T2 | 35 (83.3%) | 43 (54.4%) | 86 (61.4%) | 187 (37.8%) | 351 (46.4%) |
| T3 | 5 (11.9%) | 2 (2.5%) | 47 (33.6%) | 291 (58.8%) | 345 (45.6%) |
| T4 | 2 (4.8%) | 0 (0%) | 7 (5.0%) | 10 (2.0%) | 19 (2.5%) |
| T1 | 0 (0%) | 34 (43.0%) | 0 (0%) | 0 (0%) | 34 (4.5%) |
| unknow | 0 (0%) | 0 (0%) | 0 (0%) | 7 (1.4%) | 7 (0.9%) |

**Table S3.** Input genes for unsupervised clustering in each of the four cohorts.

| TCGA-PRAD | MSKCC | AHMU-PC | GSE25126 |
| --- | --- | --- | --- |
| HMGB2 | HMGB2 | HMGB2 | HMGB2 |
| EOMES | EOMES | EOMES | LTK |
| LTK | LTK | SLC7A8 | IFI44 |
| EIF4A1 | EIF4A1 | EIF4A1 | LAG3 |
| KIAA1683 | KIAA1683 | KIAA1683 | GPR65 |
| LYAR | LYAR | LYAR | MAP3K8 |
| IFI44 | IFI44 | IFI44 | CYBA |
| CAPN12 | CAPN12 | CAPN12 | PLXND1 |
| LAG3 | LAG3 | LAG3 | HMGB1 |
| GPR65 | GPR65 | GPR65 | MFSD10 |
| MAP3K8 | MAP3K8 | MAP3K8 | SMCO4 |
| CYBA | CYBA | CYBA | TMSB4X |
| PLXND1 | PLXND1 | PLXND1 | PPARG |
| GAB3 | GAB3 | GAB3 | H2AFZ |
| CLEC12A | CLEC12A | CLEC12A | LST1 |
| HMGB1 | HMGB1 | HMGB1 | GNLY |
| MFSD10 | MFSD10 | MFSD10 | GZMM |
| SMCO4 | TMSB4X | SMCO4 | TGFB1 |
| TMSB4X | PPARG | TMSB4X | ADA |
| PPARG | H2AFZ | PPARG | SMAD7 |
| H2AFZ | ZSWIM4 | H2AFZ | SRRT |
| ZSWIM4 | LST1 | ZSWIM4 | CRTAM |
| LST1 | GNLY | LST1 | CLEC2B |
| GNLY | GZMM | GNLY | RHBDF2 |
| GZMM | TGFB1 | ITM2B | RASSF1 |
| TGFB1 | ADA | TGFB1 | EVL |
| ADA | SMAD7 | ADA | CDK5RAP1 |
| SMAD7 | SRRT | SMAD7 | ARRB2 |
| SRRT | CRTAM | SRRT | HOPX |
| CRTAM | CLEC2B | RASD1 | FTH1 |
| CLEC2B | RHBDF2 | CLEC2B | AUTS2 |
| RHBDF2 | RASSF1 | RHBDF2 | CD53 |
| RASSF1 | EVL | RASSF1 | CA2 |
| EVL | CDK5RAP1 | EVL | OSM |
| CDK5RAP1 | ARRB2 | CDK5RAP1 | RUNX3 |
| ARRB2 | ABI3 | ARRB2 | GALC |
| ABI3 | CMC1 | ABI3 | PDIA3 |
| CMC1 | HOPX | CMC1 | NUDT7 |
| HOPX | AMICA1 | HOPX | MYL9 |
| AMICA1 | H3F3B | AMICA1 | HERPUD1 |
| H3F3B | FTH1 | H3F3B | FKBP5 |
| FTH1 | C17orf89 | FTH1 | DOCK5 |
| C17orf89 | AUTS2 | C17orf89 | AIM1 |
| AUTS2 | MED30 | AUTS2 | NBL1 |
| MED30 | CD53 | MED30 | ELF1 |
| CD53 | CA2 | CD53 | FKBP11 |
| CA2 | C9orf142 | CA2 | CELF2 |
| C9orf142 | TSEN54 | C9orf142 | CD9 |
| TSEN54 | SPEF2 | TSEN54 | CTSH |
| SPEF2 | OSM | SPEF2 | PDE4D |
| OSM | RUNX3 | OSM | DPP4 |
| RUNX3 | MAEA | RUNX3 | ITM2B |
| MAEA | GALC | MAEA | SLC7A8 |
| SRSF2 | PDIA3 | SRSF2 |  |
| GALC | NUDT7 | GALC |  |
| PDIA3 | MYL9 | PDIA3 |  |
| NUDT7 | HERPUD1 | NUDT7 |  |
| MYL9 | FKBP5 | MYL9 |  |
| HERPUD1 | RNF157 | HERPUD1 |  |
| FKBP5 | DOCK5 | FKBP5 |  |
| RNF157 | AIM1 | RNF157 |  |
| DOCK5 | NBL1 | DOCK5 |  |
| AIM1 | ELF1 | AIM1 |  |
| NBL1 | FKBP11 | NBL1 |  |
| ELF1 | CD9 | ELF1 |  |
| FKBP11 | CTSH | FKBP11 |  |
| CELF2 | PDE4D | CELF2 |  |
| CD9 | DPP4 | CD9 |  |
| CTSH | FAM177A1 | CTSH |  |
| PDE4D | RASD1 | PDE4D |  |
| DPP4 | ITM2B | DPP4 |  |
| FAM177A1 | SLC7A8 | FAM177A1 |  |
| RASD1 |  |  |  |
| ITM2B |  |  |  |
| SLC7A8 |  |  |  |

**Table S4.** The different gene mutation patterns between TregP and TregR phenotypes.

| **Genes** | **TMB** | **TregP** | **TregR** | **pvalue** | **padj** |
| --- | --- | --- | --- | --- | --- |
| TP53 | 56 (11%) | 11 (5.0%) | 45 (16.5%) | 5.07E-05 | 2.99E-03 |
| SPOP | 55 (11%) | 27 (12.4%) | 28 (10.3%) | 4.75E-01 | 9.29E-01 |
| TTN | 55 (11%) | 21 (9.6%) | 34 (12.5%) | 3.88E-01 | 8.47E-01 |
| MUC16 | 30 (6%) | 12 (5.5%) | 18 (6.6%) | 7.06E-01 | 1.00E+00 |
| FOXA1 | 29 (6%) | 10 (4.6%) | 19 (7.0%) | 3.36E-01 | 8.47E-01 |
| KMT2D | 28 (6%) | 12 (5.5%) | 16 (5.9%) | 1.00E+00 | 1.00E+00 |
| KMT2C | 26 (5%) | 10 (4.6%) | 16 (5.9%) | 5.51E-01 | 9.29E-01 |
| SPTA1 | 24 (5%) | 10 (4.6%) | 14 (5.1%) | 8.36E-01 | 1.00E+00 |
| SYNE1 | 23 (5%) | 8 (3.7%) | 15 (5.5%) | 3.94E-01 | 8.47E-01 |
| LRP1B | 22 (4%) | 6 (2.8%) | 16 (5.9%) | 1.24E-01 | 6.12E-01 |
| CSMD3 | 21 (4%) | 9 (4.1%) | 12 (4.4%) | 1.00E+00 | 1.00E+00 |
| ATM | 20 (4%) | 8 (3.7%) | 12 (4.4%) | 8.19E-01 | 1.00E+00 |
| OBSCN | 20 (4%) | 5 (2.3%) | 15 (5.5%) | 1.06E-01 | 6.12E-01 |
| FAT3 | 18 (4%) | 8 (3.7%) | 10 (3.7%) | 1.00E+00 | 1.00E+00 |
| PTEN | 18 (4%) | 7 (3.2%) | 11 (4.0%) | 8.10E-01 | 1.00E+00 |
| RYR2 | 18 (4%) | 11 (5.0%) | 7 (2.6%) | 1.57E-01 | 6.12E-01 |
| HMCN1 | 16 (3%) | 7 (3.2%) | 9 (3.3%) | 1.00E+00 | 1.00E+00 |
| MALAT1 | 15 (3%) | 7 (3.2%) | 8 (2.9%) | 1.00E+00 | 1.00E+00 |
| USH2A | 15 (3%) | 4 (1.8%) | 11 (4.0%) | 1.93E-01 | 6.12E-01 |
| CACNA1E | 15 (3%) | 4 (1.8%) | 11 (4.0%) | 1.93E-01 | 6.12E-01 |
| COL11A1 | 15 (3%) | 4 (1.8%) | 11 (4.0%) | 1.93E-01 | 6.12E-01 |
| RYR1 | 15 (3%) | 3 (1.4%) | 12 (4.4%) | 6.51E-02 | 5.49E-01 |
| MUC17 | 15 (3%) | 2 (0.9%) | 13 (4.8%) | 1.58E-02 | 3.11E-01 |
| MYO15A | 14 (3%) | 5 (2.3%) | 9 (3.3%) | 5.92E-01 | 9.70E-01 |
| MACF1 | 13 (3%) | 6 (2.8%) | 7 (2.6%) | 1.00E+00 | 1.00E+00 |
| ZFHX3 | 13 (3%) | 4 (1.8%) | 9 (3.3%) | 4.02E-01 | 8.47E-01 |
| RP1 | 13 (3%) | 9 (4.1%) | 4 (1.5%) | 9.00E-02 | 6.12E-01 |
| NALCN | 13 (3%) | 2 (0.9%) | 11 (4.0%) | 4.50E-02 | 4.74E-01 |
| FBN1 | 12 (2%) | 5 (2.3%) | 7 (2.6%) | 1.00E+00 | 1.00E+00 |
| CSMD1 | 12 (2%) | 6 (2.8%) | 6 (2.2%) | 7.73E-01 | 1.00E+00 |
| DNAH17 | 12 (2%) | 6 (2.8%) | 6 (2.2%) | 7.73E-01 | 1.00E+00 |
| ABCA13 | 12 (2%) | 3 (1.4%) | 9 (3.3%) | 2.41E-01 | 7.11E-01 |
| DCHS2 | 12 (2%) | 1 (0.5%) | 11 (4.0%) | 1.50E-02 | 3.11E-01 |
| CTNNB1 | 11 (2%) | 4 (1.8%) | 7 (2.6%) | 7.62E-01 | 1.00E+00 |
| GPR98 | 11 (2%) | 4 (1.8%) | 7 (2.6%) | 7.62E-01 | 1.00E+00 |
| BAI3 | 11 (2%) | 6 (2.8%) | 5 (1.8%) | 5.50E-01 | 9.29E-01 |
| RYR3 | 11 (2%) | 6 (2.8%) | 5 (1.8%) | 5.50E-01 | 9.29E-01 |
| GRIA1 | 11 (2%) | 3 (1.4%) | 8 (2.9%) | 3.60E-01 | 8.47E-01 |
| HECTD4 | 10 (2%) | 4 (1.8%) | 6 (2.2%) | 1.00E+00 | 1.00E+00 |
| NAV2 | 10 (2%) | 4 (1.8%) | 6 (2.2%) | 1.00E+00 | 1.00E+00 |
| DST | 10 (2%) | 4 (1.8%) | 6 (2.2%) | 1.00E+00 | 1.00E+00 |
| FLG | 10 (2%) | 4 (1.8%) | 6 (2.2%) | 1.00E+00 | 1.00E+00 |
| FBN3 | 10 (2%) | 4 (1.8%) | 6 (2.2%) | 1.00E+00 | 1.00E+00 |
| KDM6A | 10 (2%) | 5 (2.3%) | 5 (1.8%) | 7.57E-01 | 1.00E+00 |
| PCLO | 10 (2%) | 5 (2.3%) | 5 (1.8%) | 7.57E-01 | 1.00E+00 |
| APC | 10 (2%) | 5 (2.3%) | 5 (1.8%) | 7.57E-01 | 1.00E+00 |
| EPB41L3 | 10 (2%) | 3 (1.4%) | 7 (2.6%) | 5.23E-01 | 9.29E-01 |
| CFH | 10 (2%) | 3 (1.4%) | 7 (2.6%) | 5.23E-01 | 9.29E-01 |
| SALL1 | 10 (2%) | 3 (1.4%) | 7 (2.6%) | 5.23E-01 | 9.29E-01 |
| CNTNAP5 | 10 (2%) | 6 (2.8%) | 4 (1.5%) | 3.52E-01 | 8.47E-01 |
| TNXB | 10 (2%) | 6 (2.8%) | 4 (1.5%) | 3.52E-01 | 8.47E-01 |
| XIRP2 | 10 (2%) | 6 (2.8%) | 4 (1.5%) | 3.52E-01 | 8.47E-01 |
| ZMYM3 | 10 (2%) | 2 (0.9%) | 8 (2.9%) | 1.97E-01 | 6.12E-01 |
| STAB2 | 10 (2%) | 2 (0.9%) | 8 (2.9%) | 1.97E-01 | 6.12E-01 |
| GAD2 | 10 (2%) | 2 (0.9%) | 8 (2.9%) | 1.97E-01 | 6.12E-01 |
| CACNA1A | 10 (2%) | 2 (0.9%) | 8 (2.9%) | 1.97E-01 | 6.12E-01 |
| RNF213 | 10 (2%) | 2 (0.9%) | 8 (2.9%) | 1.97E-01 | 6.12E-01 |
| MXRA5 | 10 (2%) | 1 (0.5%) | 9 (3.3%) | 4.82E-02 | 4.74E-01 |
| PIK3CA | 10 (2%) | 1 (0.5%) | 9 (3.3%) | 4.82E-02 | 4.74E-01 |

**SUPPLEMENTARY FIGURES**


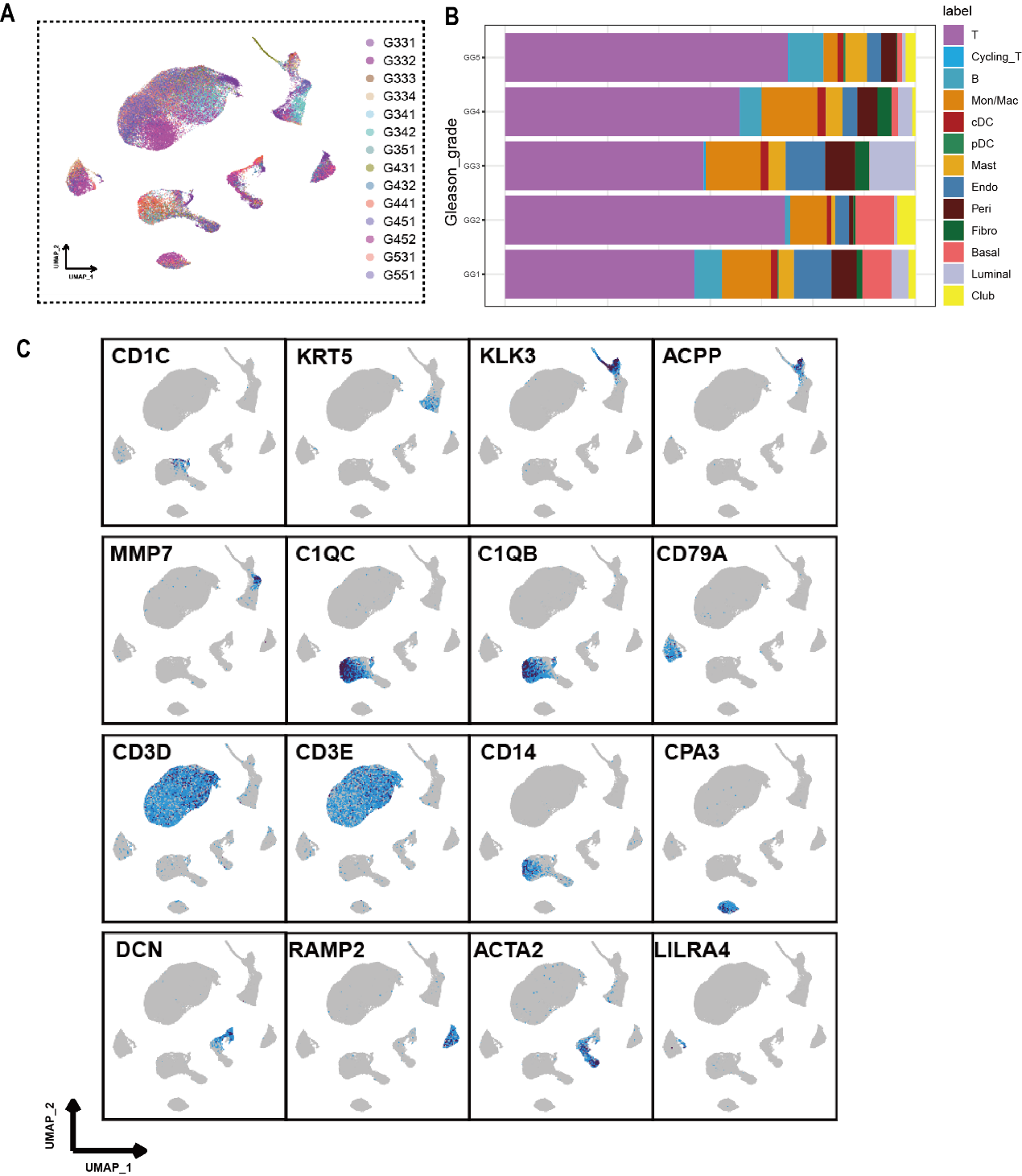


**Fig. S1: Uniform Manifold Approximation and Projection (UMAP) visualization:** (**A**) UMAP visualization of 14 tumor samples; (**B**) The proportions of 13 cell types among five Gleason groups; (**C**) The expression of marker genes of the 13 cell types.


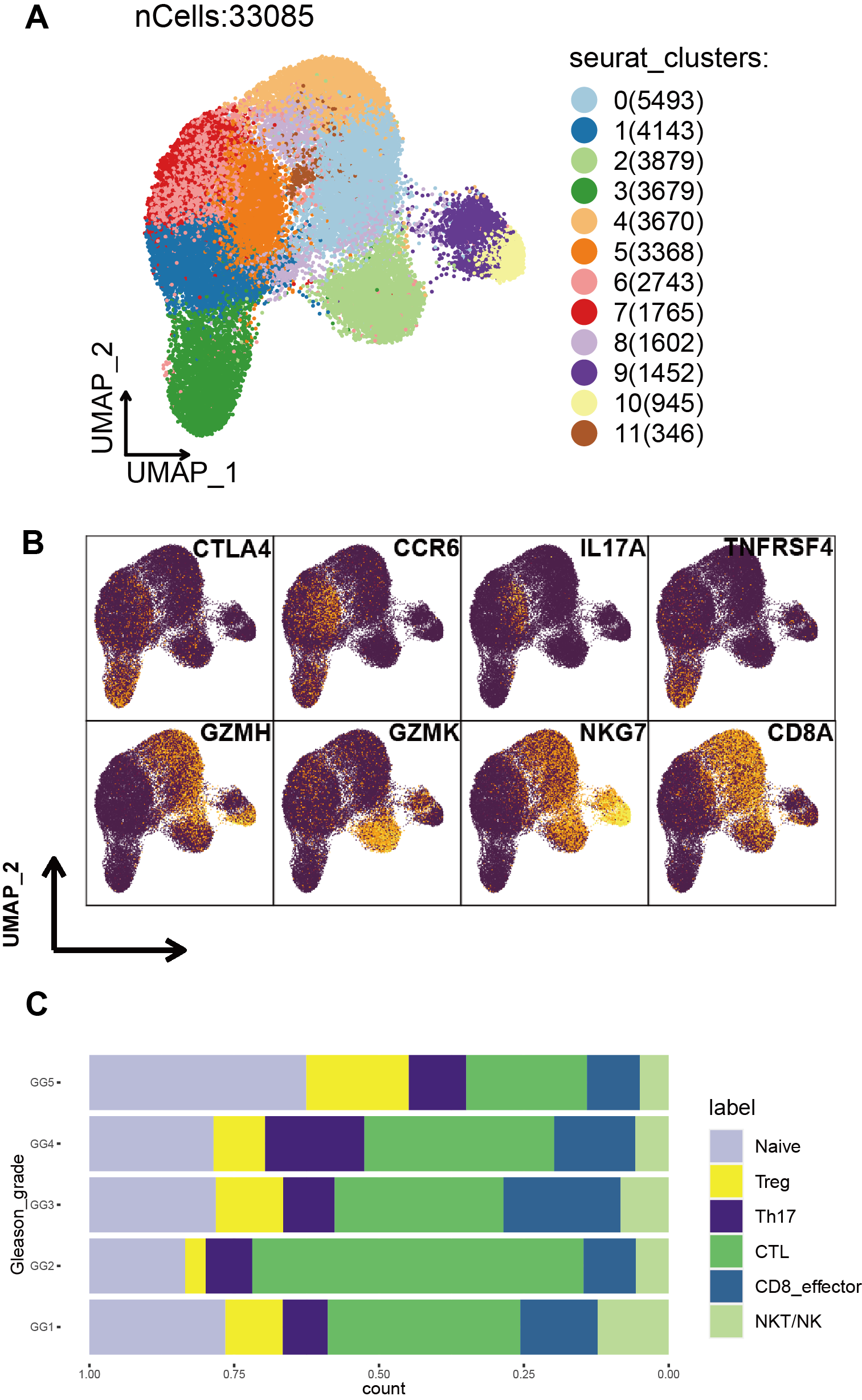


**Fig. S2: Identification of six T cell subpopulations.** (**A**) UMAP plot of 11 clusters; (**B**) UMAP plot of expression of specific T cell markers; (**C**) Distribution of these six type T cells in different Gleason groups. There were highly variable T cell infiltration profiles in different Gleason groups of prostate cancer (PCa), especially Treg cells and Th17 cells.


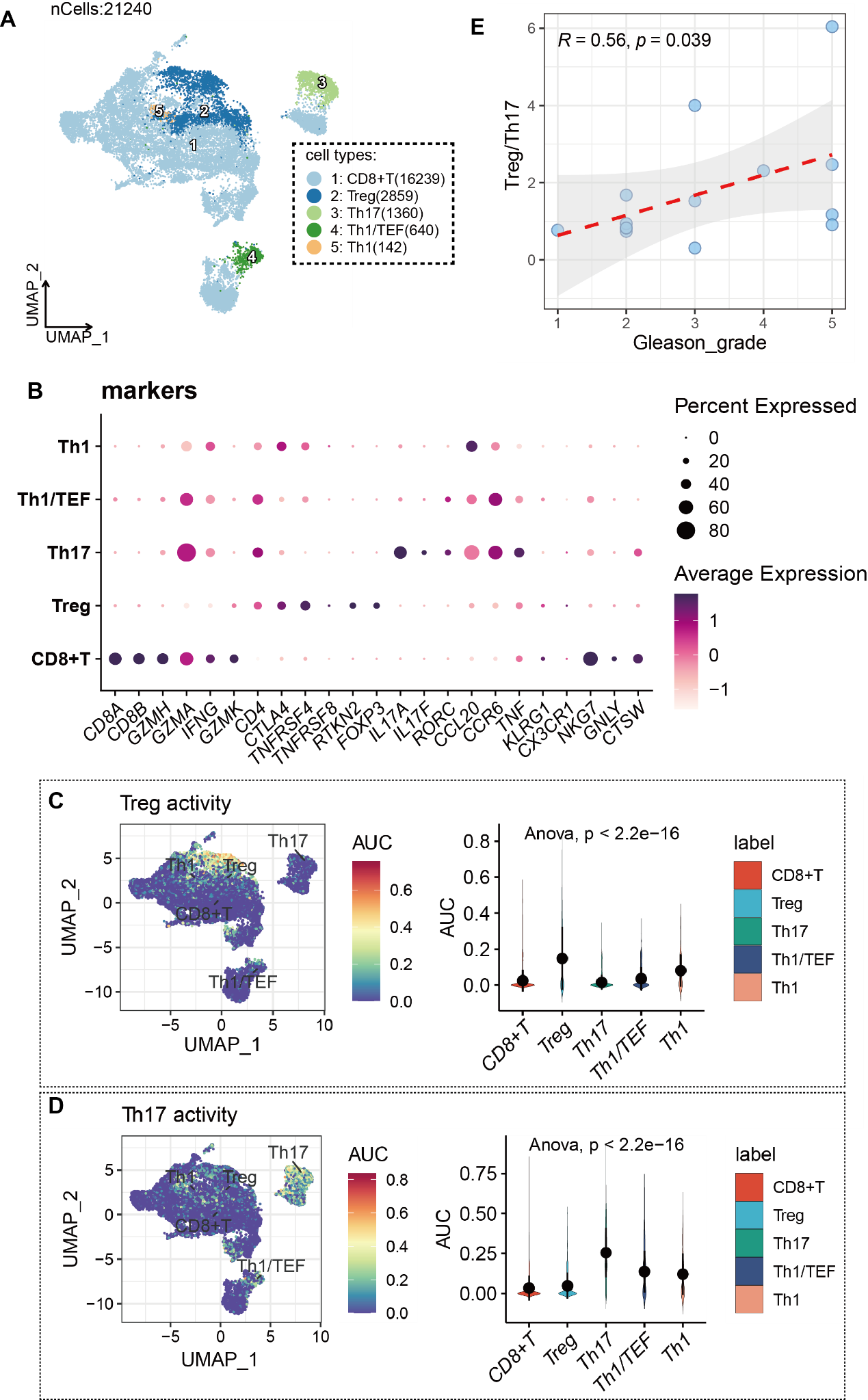


**Fig. S3: Validation in external scRNA-sequencing cohort.** (**A**) UMAP visualization of 21240 T cells, including CD8+T(n=16239), Treg(n=2859), Th17(n=1360), Th1/TEF(n=640), Th1(n=142); (**B**) Dotplot showed expression of signature genes among T cells, including CD8+T (*CD8A, CD8B, GZMH, GZMA, IFNG, GZMK*), Treg(*CD4, CTLA4, TNFRSF4, TNFRSF8, RTKN2, FOXP3*), Th17 (*CD4*, *IL17A, IL17F, RORC, CCL20, CCR6*), Th1(*CD4, TNF*), TEF(*KLRG1, CX3CR1, NKG7, GNLY, CTSW*); (**C**) UMAP plot showed the distribution of Treg activity scores among T cells and Treg cell presented visually higher score(left); comparison of Treg activity scores showed significantly highest scores (right, *P* < 2.2e-16), which confirmed the accuracy of annotations. (**D**) UMAP plot showed the distribution of Th17 activity scores among T cells and Treg cell presented visually higher score(left); comparison of Th17 activity scores showed significantly highest scores (right, *P* < 2.2e-16). (**E**) Correlations analysis identified the positive correlations between Treg/Th17 ratios and Gleason grades (R = 0.56, *P* = 0.039).


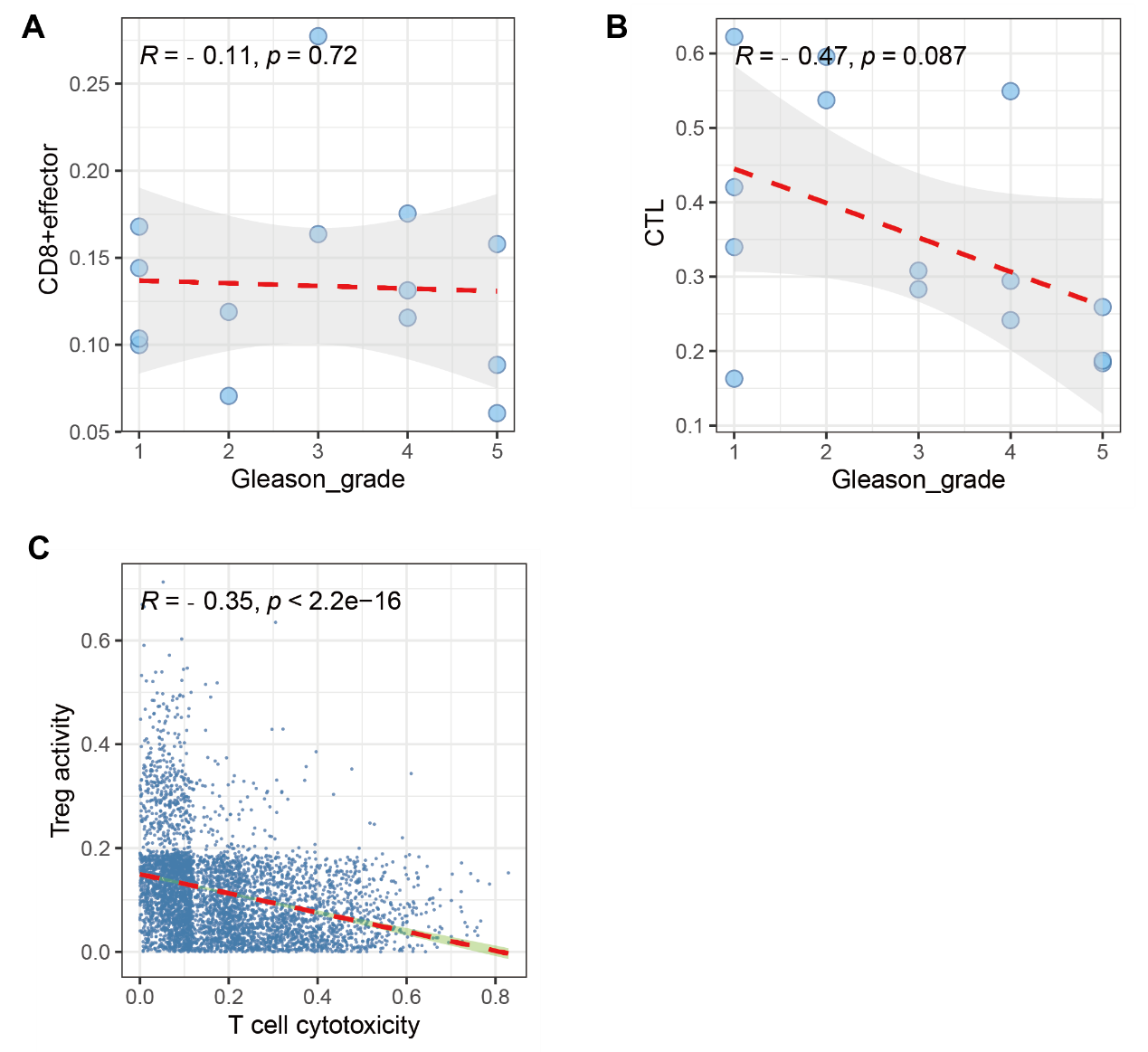


**Fig. S4: Correlation analysis between Gleason grades and CD8+effector and CTL cells, and correlation between Treg activity and T cell cytotoxicity.** (**A**) The infiltration of CD8+effector cells were negatively correlated to Gleason grades (R = -0.11, *P* = 0.72); (**B**) The infiltration of CTL were negatively correlated to Gleason grades (R = -0.47, *P* = 0.087); (**C**) Utility of AUCell algorithm calculated Treg activity and T cell cytotoxicity scores for all of the cells, and the results showed that there were significantly negative correlation between Treg activity and T cell cytotoxicity(R = 0.35, P < 2.2e-16), implicating the adverse effects of Treg cell in the process of immune cell killing tumor cells.


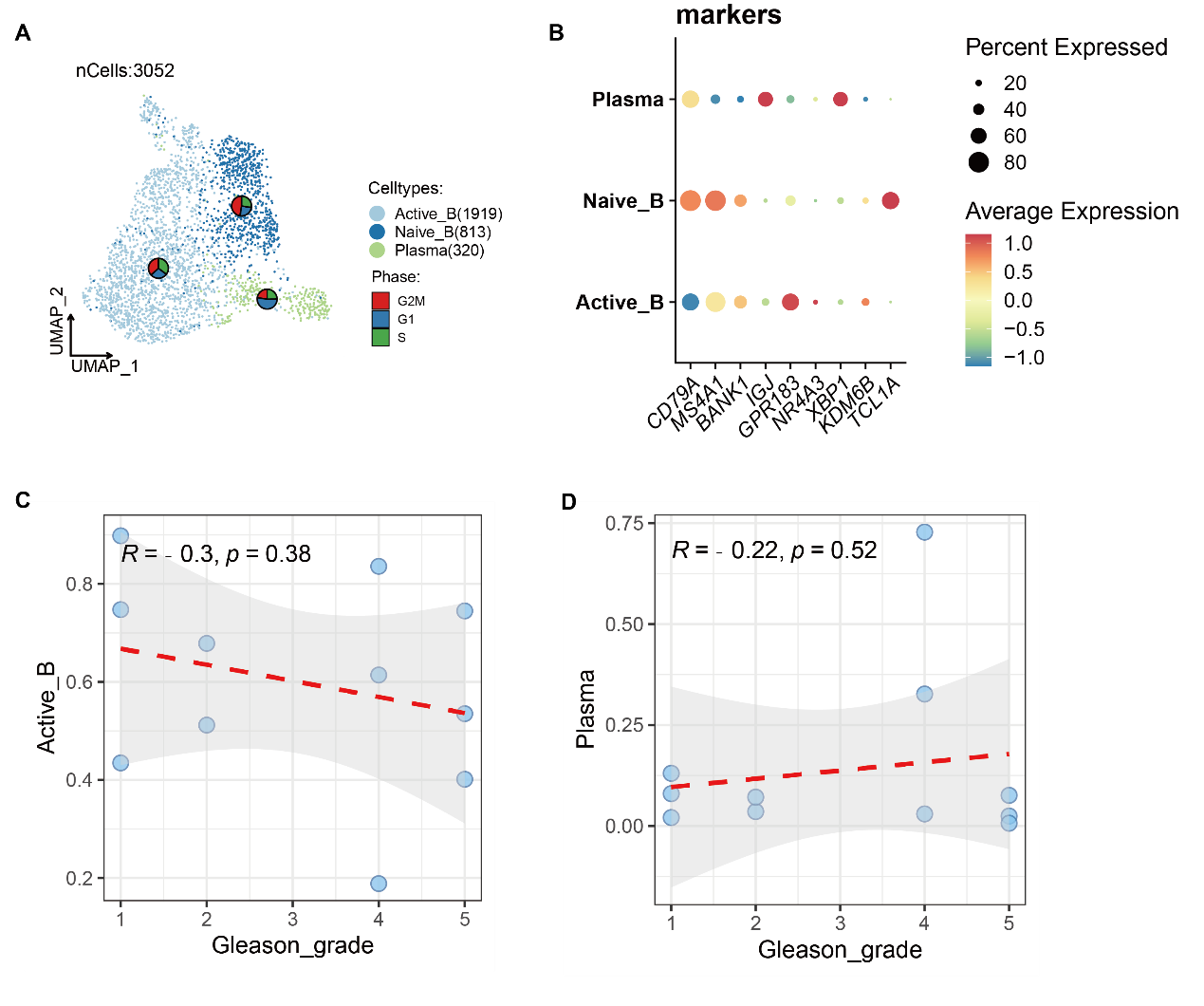


**Fig. S5: Correlation analysis between Gleason grades and B cells.** (**A**) UMAP visualization of three B cells; (**B**) Dotplot showed expression of signature genes among the three defined B cells; (**C**) Correlations between Gleason grades and Activate_B cells, and a negative correlation was described (R = -0.3, *P* = 0.38); (**D**) Correlations between Gleason grades and Plasma cells, and a negative correlation was described (R = -0.22, *P* = 0.52), while no significance.


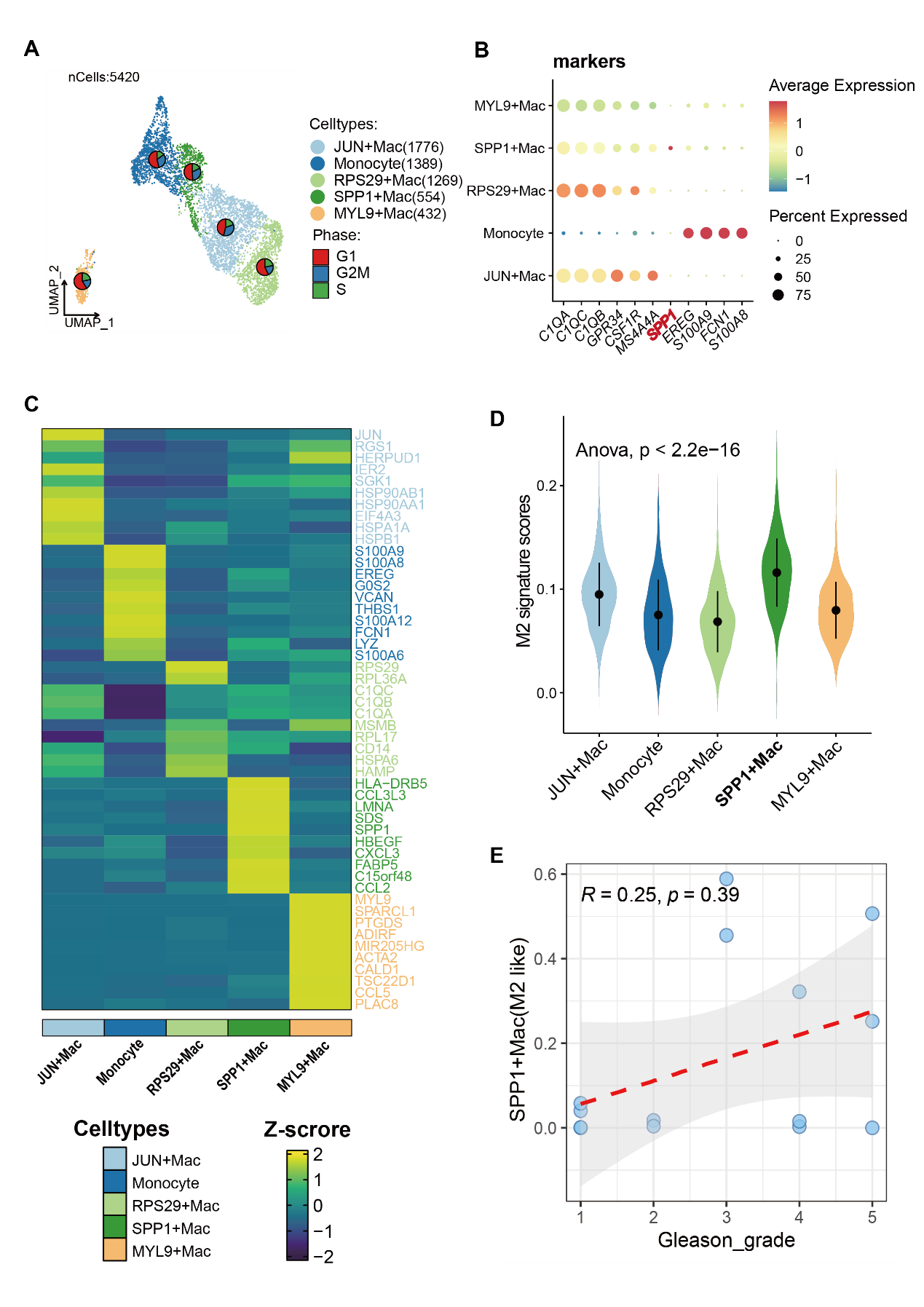


**Fig. S6: Correlation analysis between Gleason grades and SPP+ Macrophage.** (**A**) UMAP visualization of monocytes and macrophages. Mac: macrophage; (**B**) Dotplot showed expression of signature genes; (**C**) Heatmap showed the top 10 differentially expressed genes of five cell populations. (**D**) Comparison of M2 signature gene scores between macrophages and monocytes, and SPP1+Mac exhibited the highest scores (*P* <2.2e-16); (**E**) Correlations analysis revealed the infiltration of SPP1+Mac positively correlated to higher Gleason grades (R = 0.25, *P* = 0.39), indicating a poor prognosis, while not statistically significant, possibly due to the extreme value in Gleason grade 3.


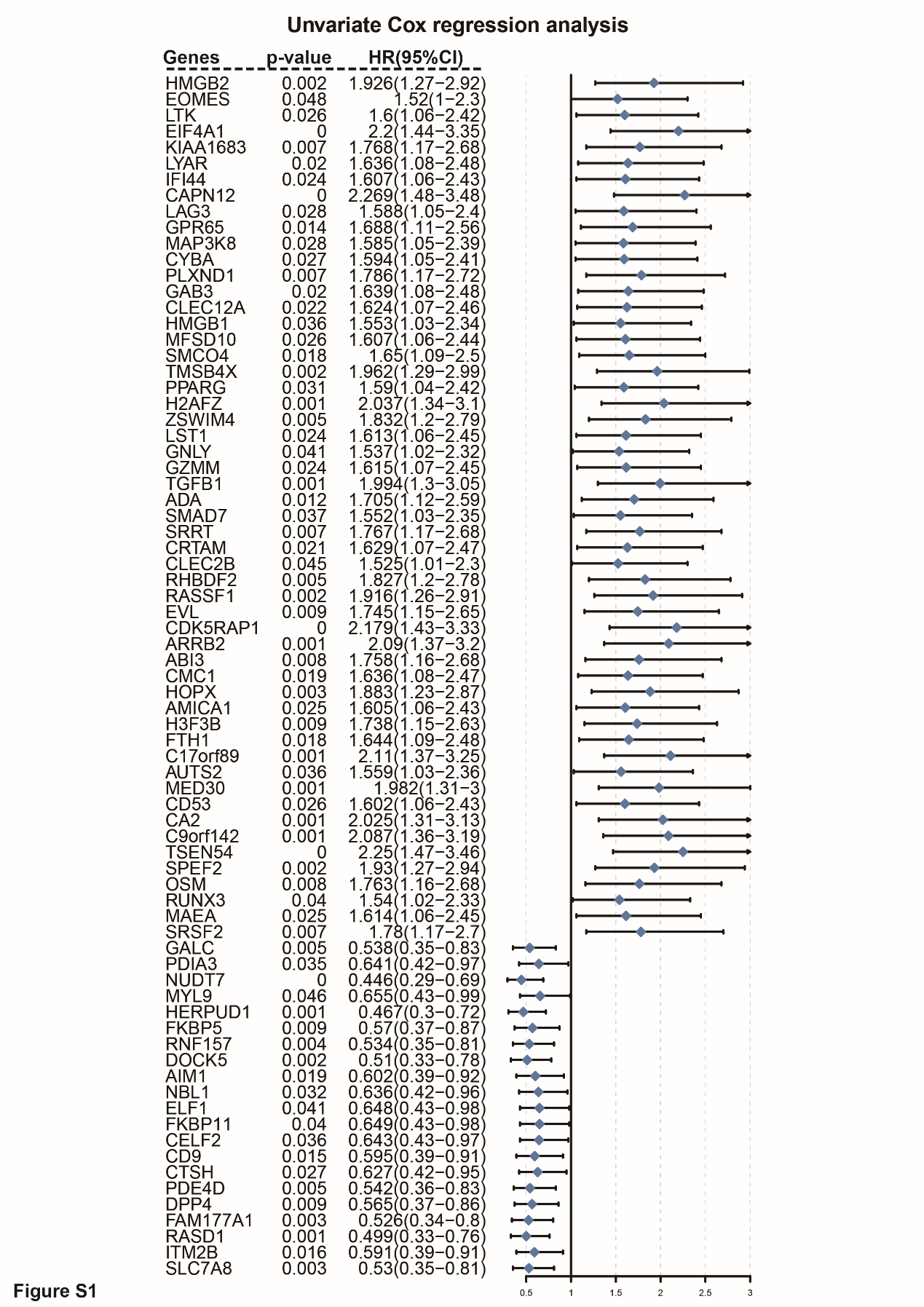


**Fig. S7: Seventy-five branch-dependent genes filtrated by univariate Cox regression analysis.**


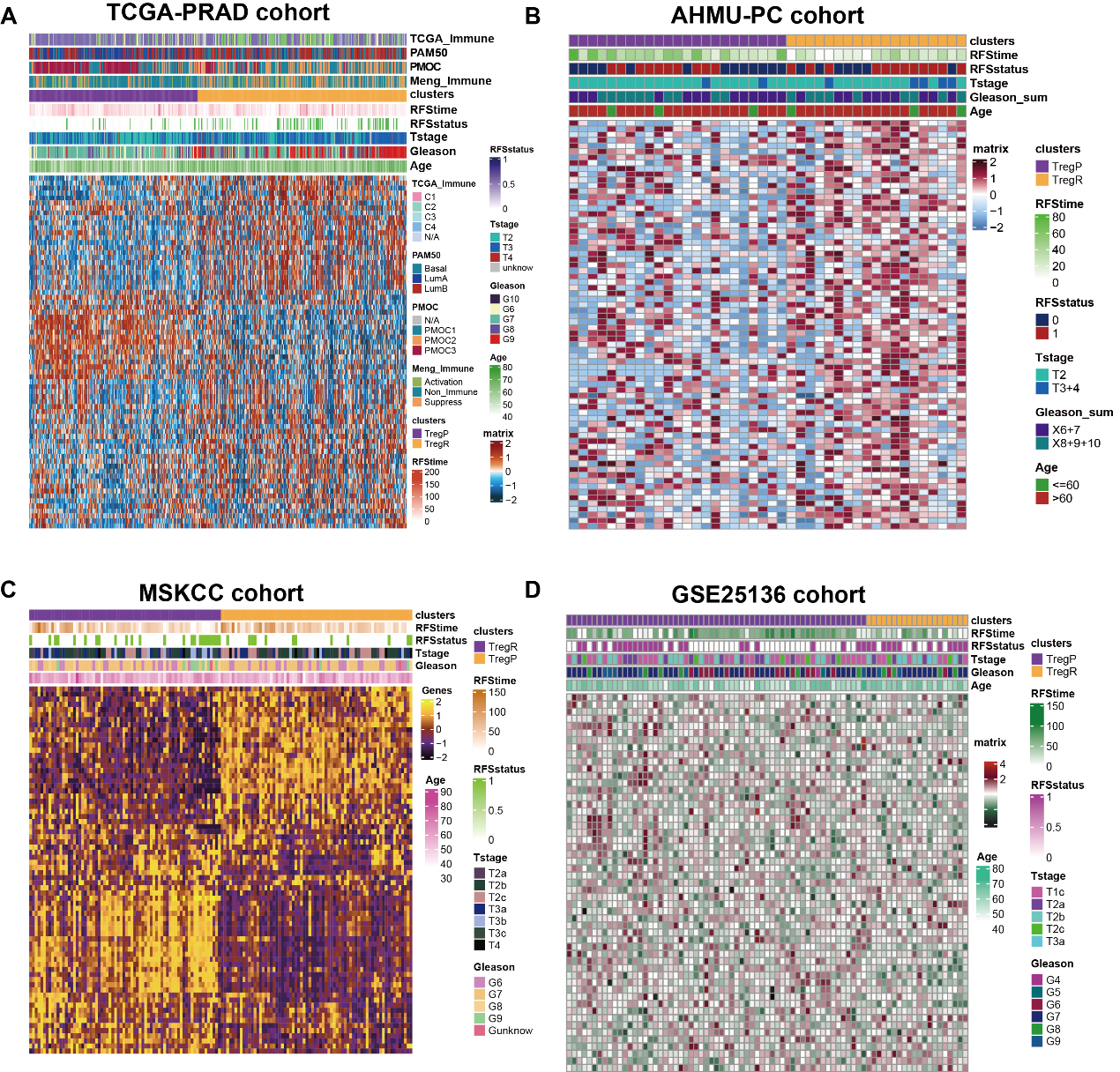


**Fig. S8:** **Distribution of model-construction genes among TregR and TregP subtypes.** (**A**) Heatmap showed the expression of 75 genes between TregP and TregR clusters in TCGA-PRAD cohort; (**B**) Heatmap showed the expression of 72 genes between TregP and TregR clusters in AHMU-PC cohort; (**C**) Heatmap showed the expression of 72 genes between TregP and TregR clusters in MSKCC cohort; (**D**) Heatmap showed the expression of 53 genes between TregP and TregR clusters in GSE25136 cohort.


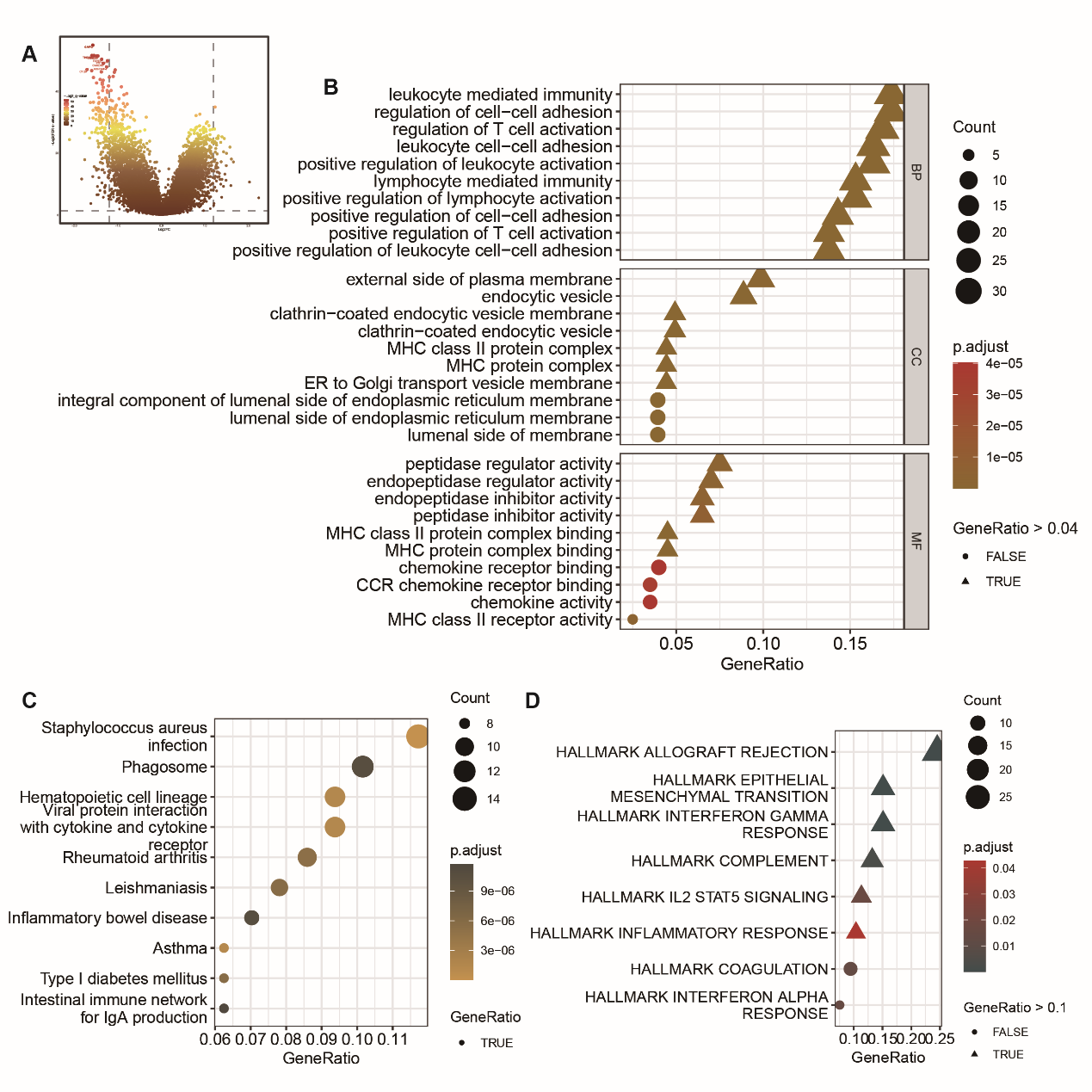
**Fig. S9:** **Different activated pathways between TregP and TregR PCa.** (**A**) The volcano diagram shows 215 differentially expressed genes between TregR and TregP subtypes; (**B**) GO enrichment analysis (BP: Biological Process; CC: Cellular Component; MF: Molecular Function), and the top 10 items were visualized; (**C**) KEGG enrichment analysis; (**D**) HALLMARK analysis.


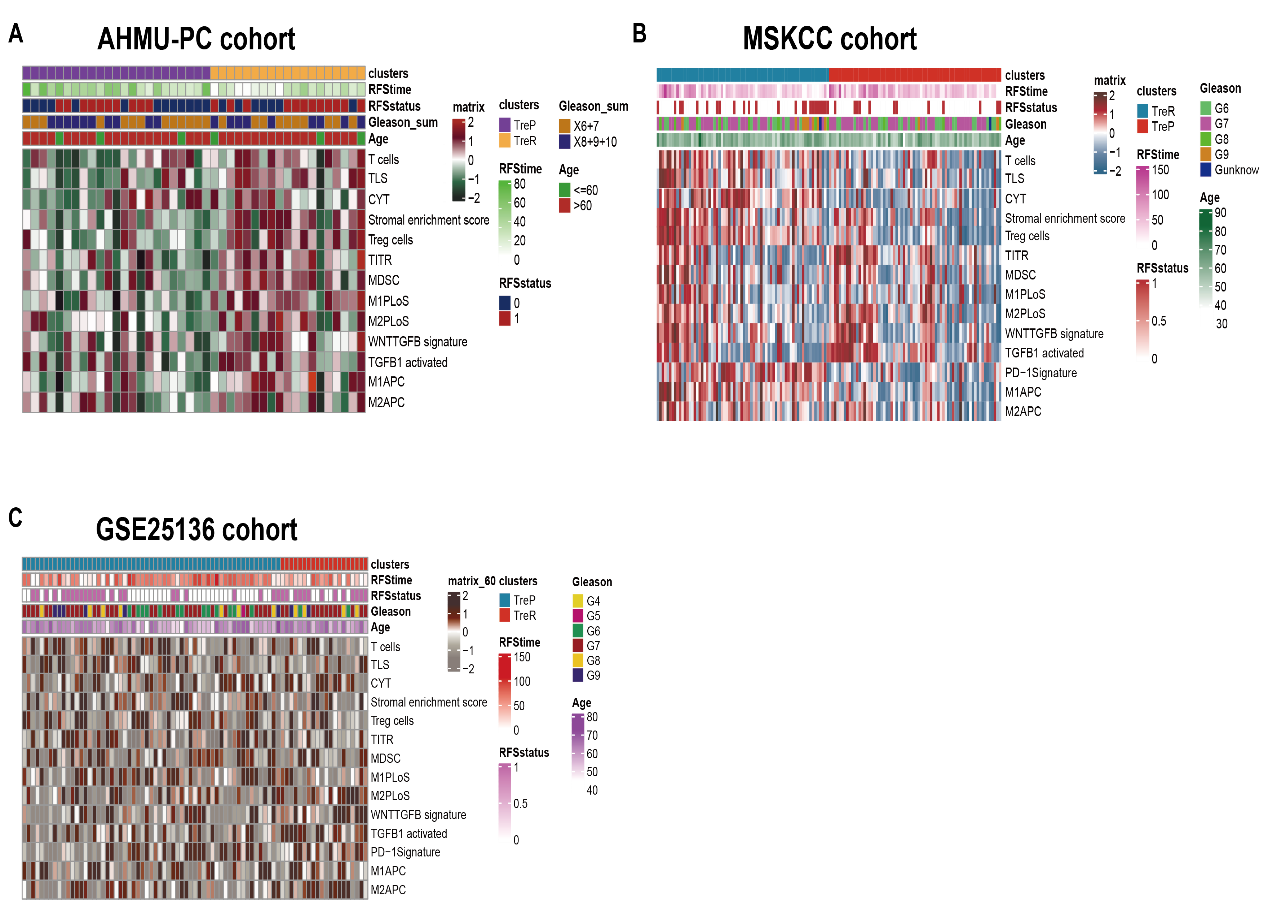


**Fig. S10: Assessment of tumor microenvironment status among TregP and TregR subtypes in three external cohorts**. (**A**) The distribution of 13 immune-relevant activation scores of the two subtypes in AHMU-PC cohort, TregR also exhibited more obvious feature of immune suppression, such as higher scores of TLS, TITR, MDSC, WNT/TGF-β, TGF-β1 activated; (**B**) The distribution of 14 immune-relevant activation scores of the two subtypes in MSKCC cohort, TregR exhibited higher scores of TLS, stromal enrichment score, Treg, TITR, MDSC, WNT/TGF-β, and TGF-β1 than TregP; (**C**) The distribution of 14 immune-relevant activation scores of the two subtypes in GSE25136 cohort, and TregR had higher scores of WNT/TGF-β and TGF-β1 activated.


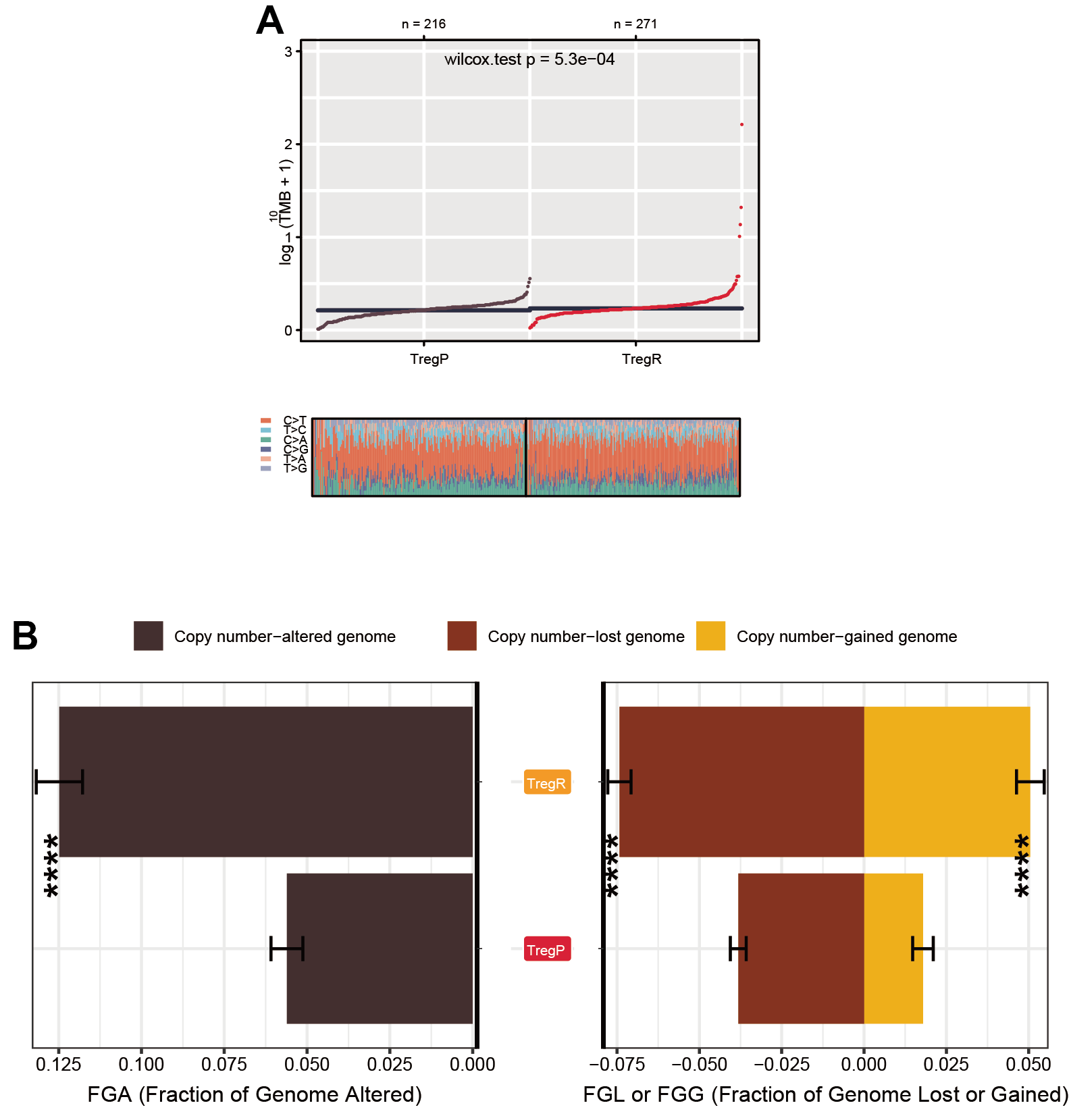


**Fig. S11: Comparison of gene mutation patterns.** (**A**) Comparison of tumor mutation burden between the two subtypes; (**B**) Different genome alterations of the two subtypes, including faction of genome altered, fraction of genome lost or gained.


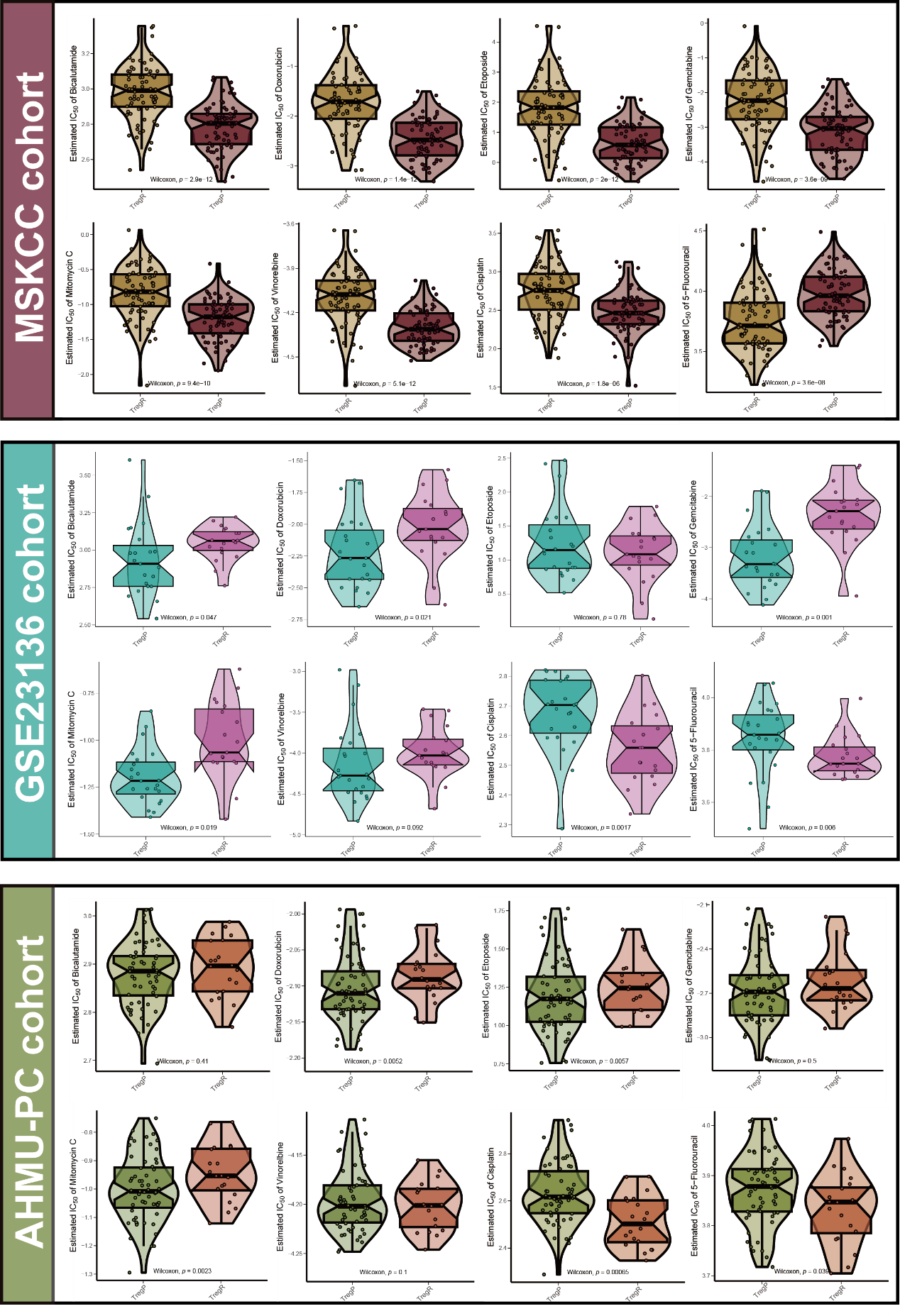


**Fig. S12: Comparison of sensitivity to eight chemicals between TregR group and TregP group in three external cohorts.** Comparison of sensitivity to eight chemicals between TregR group and TregP group in MSKCC cohort, TregP exhibited higher susceptibility to bicalutamide (*P* = 2.9e-12), doxorubicin (*P* = 1.4e-12), etoposide (*P* = 2e-12), gemcitabine (*P* = 3.6e-09), mitomycin C (*P* = 9.4e-10), vinorelbine (*P* = 5.1e-12), and cisplatin (*P* =1.8e-06), while only 5-fluorouracil is suitable for TregR (*P* = 3.6e-08)(**upper**); Comparison of the Treg activity between C1 and C2 clusters in GSE25136 cohort, TregP exhibited higher susceptible to bicalutamide (*P* = 0.41), doxorubicin (*P* = 0.0052), etoposide (*P* = 0.0057), gemcitabine (*P* = 0.5), mitomycin C (*P* = 0.0023), and vinorelbine (*P* = 0.1), while TregR was more sensitive to 5-fluorouracil (*P* =0.039) and cisplatin (*P* =0.00065) (**middle**); Comparison of sensitivity to eight chemicals between TregR group and TregP group in AHMU-PC cohort, TregP exhibited higher susceptible to bicalutamide (*P* = 0.047), doxorubicin (*P* = 0.021), gemcitabine (*P* = 0.001), mitomycin C (*P* = 0.019), and vinorelbine (*P* = 0.092), while TregR was more sensitive to 5-fluorouracil (*P* =0.006) and cisplatin (*P* =0.0017) (**lower**).


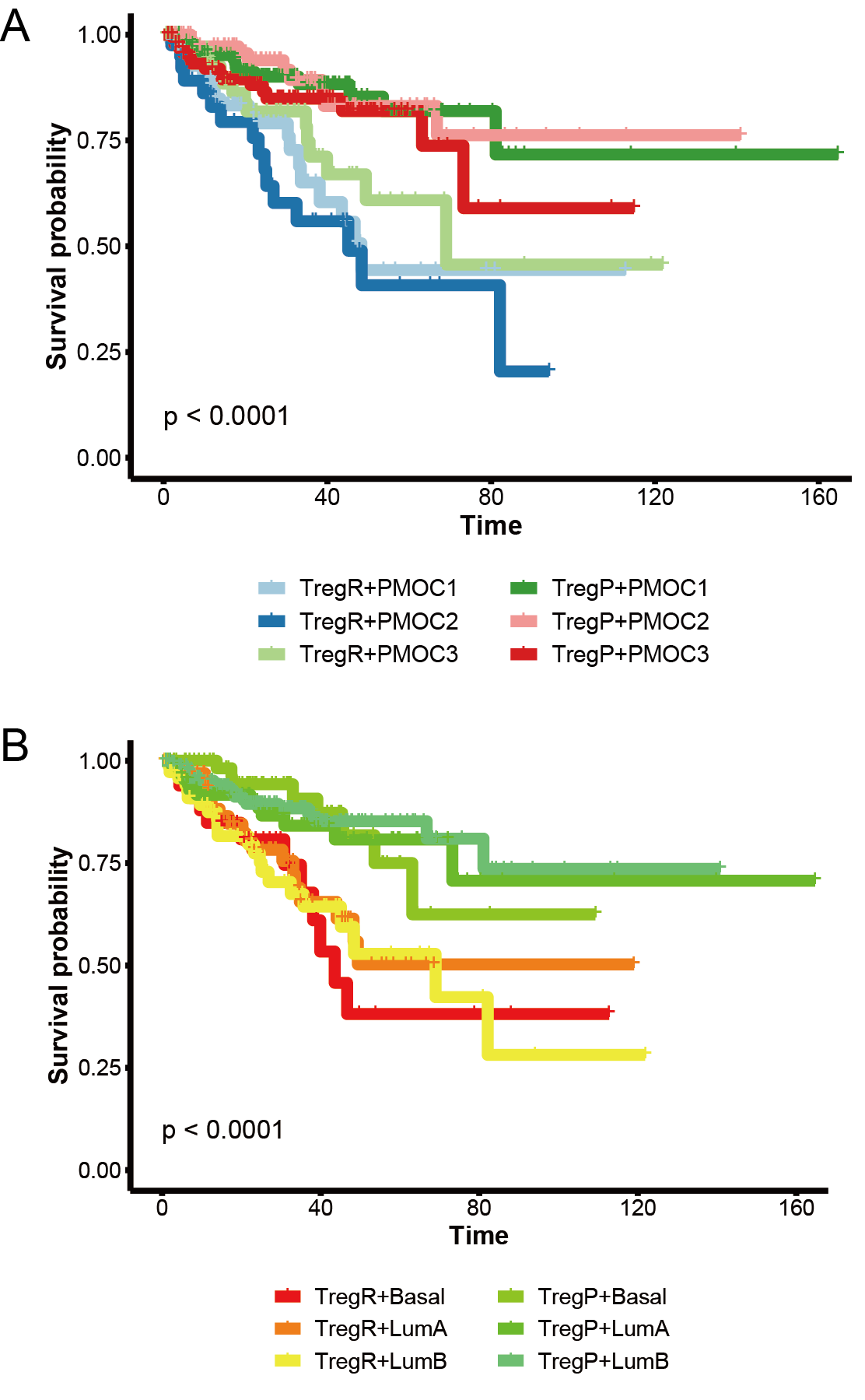


**Fig. S13: Survival analysis for the six newly defined subtypes.** (**A**) Using the PMOC and Treg-PCa typing systems to define patients in TCGA-PRAD cohort, we obtained 6 phenotypes, and K-M plot shows for the six newly defined subtypes, where TregR/PMOC2 exhibited the worst clinical outcome (*P* < 0.0001); (**B**) Through similar method mentioned above, six subtypes were defined from PAM50 and Treg-PCa classifications, and K-M plot showed that TregR /luminal B represented the poorest phenotype (*P* < 0.0001).
